# Latexin deletion in the bone marrow niche suppresses leukemia via LIFR downregulation and immune activation

**DOI:** 10.1172/jci.insight.199771

**Published:** 2026-10-08

**Authors:** Cuiping Zhang, Xia Yao, Pinpin Sui, Liming Hou, Bowen Yan, Larry L. Luchsinger, Gang Huang, Sheng Tong, Youwen Zhang, Bojing Shao, Hong Qian, Hong Zheng, Hui Zhong, Feng-Chun Yang, Ying Liang

**Affiliations:** 1 Department of Toxicology and Cancer Biology, University of Kentucky, Lexington, Kentucky, USA.; 2 Lindsley F. Kimball Research Institute, New York Blood Center, Rye, New York, USA.; 3 Department of Cell Systems & Anatomy, University of Texas Health at San Antonio, San Antonio, Texas, USA.; 4 Department of Pharmacodynamics, College of Pharmacy, University of Florida, Gainesville, Florida, USA.; 5 Department of Bioengineering, University of Kentucky, Lexington, Kentucky, USA.; 6 Center for Computational and Integrative Biology, Rutgers University-Camden, Camden, New Jersey, USA.; 7 Karolinska Institute, Flemingsberg Campus, Huddinge, Sweden.; 8 Division of Hematology/Oncology, Penn State University College of Medicine, Hershey, Pennsylvania, USA.

**Keywords:** Hematology, Oncology, Leukemias

## Abstract

Acute myeloid leukemia (AML) is the most common adult leukemia diagnosis. Bone marrow (BM) niche significantly influences the initiation and progression of AML. However, our knowledge about the effect of leukemic niche on leukemia stem cells (LSC) and leukemogenesis is limited. In this study, we identified an extrinsic-regulatory function of latexin (*Lxn*) in leukemogenesis. Using a MLL-AF9–induced AML mouse model in WT and *Lxn*-KO (*Lxn^–/–^*) recipient mice, we found that *Lxn* deletion in the BM niche enhanced the survival of AML mice by suppressing LSCs and reducing blood blasts. Single-cell RNA-seq of stromal cells and cell communication analysis uncovered downregulation of the leukemia inhibitory factor receptor (LIFR) signaling pathway in the *Lxn^–/–^* niche, particularly within mesenchymal stromal cells (MSCs). Mechanistically, reduced LIFR level in Lxn*^–/–^* MSCs upregulated *Cxcl9* expression, leading to increased recruitment of CD8 T cells and enhanced cytotoxicity against leukemic cells. Combination of *Lxn* niche deletion and immune checkpoint inhibitor PD-1 further prolonged survival. The findings have important clinical implications, suggesting that *Lxn* inhibition could improve the efficacy of AML therapies by targeting the leukemia niche and enhancing immune surveillance.

## Abstract 2

This study reveals how the bone marrow leukemic niche regulates leukemia development and shapes antileukemic immune responses.

## Graphical abstract

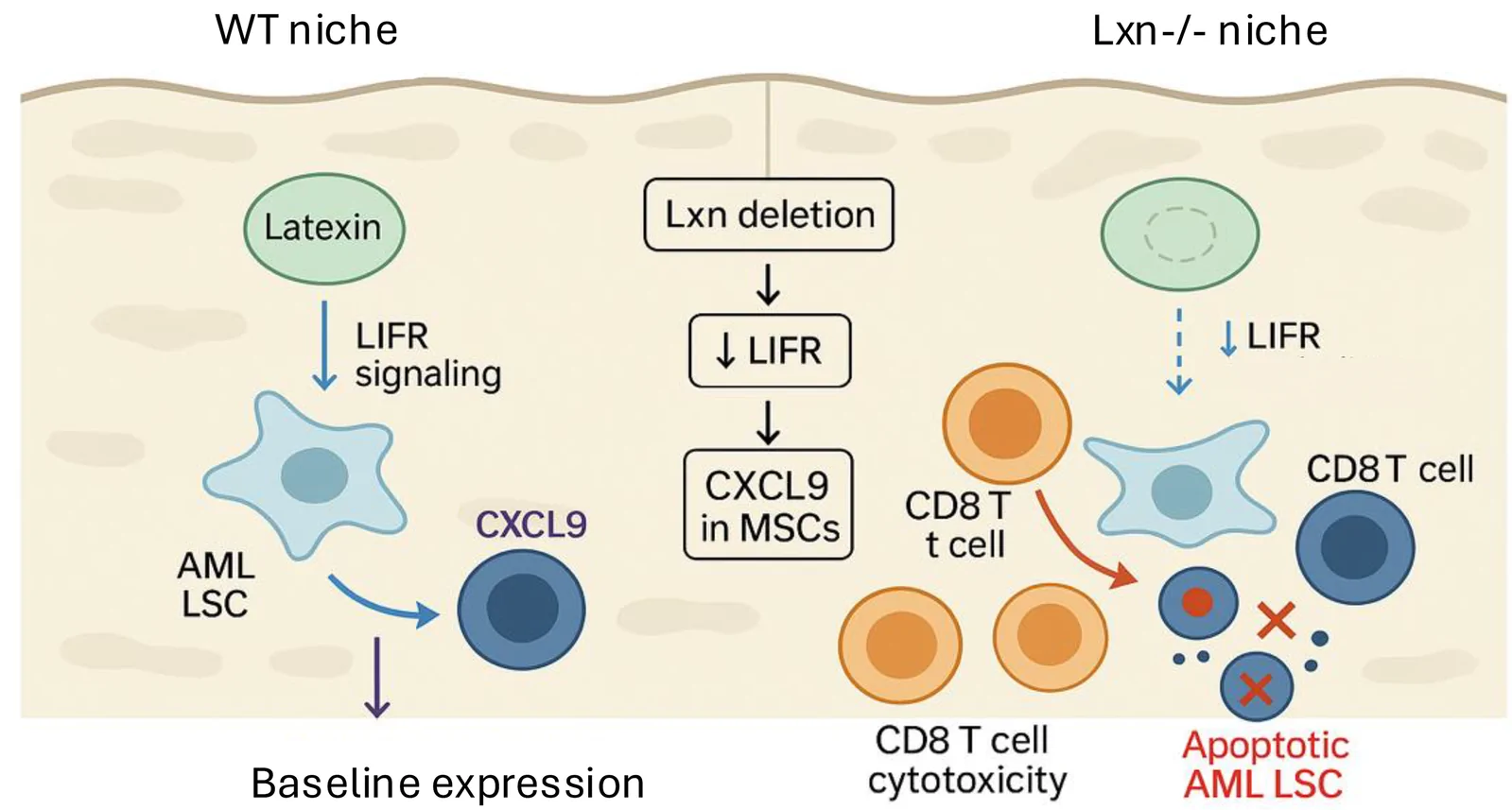

## Introduction

Acute myeloid leukemia (AML) is the most common adult leukemia with a dismal survival rate among all subtypes of leukemia (<50% 5-year overall survival rate) (1). It is characterized by excessive proliferation of abnormal and immature leukemic cells that are the main targets of current treatment regimens. Such limit causes the key challenges of AML therapy, such as relapse, refractory disease, and malignancy-associated morbidity and mortality (2). Thus, there is a pressing need to develop new therapeutic strategies.

Genetic and epigenetic alterations could lead to AML initiation and progression (3). Among them, chromosomal translocations involving the mixed lineage leukemia gene (*MLL*) give rise to highly aggressive AML associated with poor clinical outcomes (4). The most common translocation is t(9;11), which encodes the oncogenic MLL-AF9 (MA9) fusion protein (5). In AML, the bone marrow (BM) microenvironment undergoes profound changes, becoming a supportive niche for leukemic cell survival and proliferation while disrupting normal hematopoiesis (6). Recent findings at the single-cell level have provided significant insights into how AML remodels the BM niche (7–14). Key characteristics of the AML-altered niche include (a) impaired mesenchymal stromal cells (MSCs) with decreased ability to support normal hematopoiesis (15, 16); (b) diminished osteogenic potential of MSCs resulting in reduced osteoblast numbers and bone formation (17); (c) vascular and endothelial changes disrupting normal blood supply and oxygenation (18); (d) metabolic alteration favoring AML cell survival (19); and (e) inflammation and immune suppression (11).

The role of niche in AML development is further shown in mouse genetic models in which deletion or activation of specific genes in stromal cells could lead to a variety of hematopoietic malignancies. For example, deletion of *Lama4* in MSC accelerates AML onset and confers chemoresistance (20). Deletion of *Dicer1* or *Sbds* gene in MSCs causes myelodysplastic syndrome-like disease with sporadic transformation to AML (21). Deletion of *IκBα*, *Rarg*, *Rb1*, *Mib1*, *Sipa1*, or *Crebbp* in stromal cells leads to myeloproliferative neoplasm, while activation of *β-catenin* in osteoblasts and *Ptpn11* in MSCs causes AML (22–28). Moreover, patients with AML show the presence of genetic alterations in MSCs, albeit of different oncogenic abnormalities present in the leukemic blasts (29, 30). These studies suggest that genetic and functional alterations in niche cells contribute to leukemogenesis. However, far less is known about the underlying molecular mechanism, which may limit niche as a potential therapeutic target for AML treatment.

We previously identified the cell-intrinsic regulatory role of latexin (*Lxn*) in hematopoiesis (31–33) and have published studies uncovering functions and mechanisms of *Lxn* in both normal and stress hematopoiesis (34–41). Specifically, we demonstrated that *Lxn* deletion: (a) expands HSC with enhanced self-renewal and long-term repopulating capacity; (b) protects HSCs from radiation- and chemotherapy-induced damages and mitigates myelosuppression; and (c) rejuvenates old HSCs by increasing self-renewal and balancing lineage differentiation. Mechanistically, we found that (d) *Lxn* deletion downregulates thrombospondin 1 (*Thbs1*) in homeostatic condition, thus increasing HSC self-renewal; and (e) under radiation and 5-FU stresses, *Lxn* deletion upregulates *Bcl-2*, enhancing HSC survival. Recently, we reported that (f) *Lxn* regulates male and female hematopoiesis differently in homeostatic condition. Furthermore, we studied its transcriptional regulation and downstream signaling pathways, and we found that (g) ribosomal protein subunit 3 (RPS3) is a Lxn binding protein and (h) high-mobility group protein 2 (HMGB2) is a transcription suppressor of *Lxn* in HSCs. Taken together, our work establishes Lxn as a key cell-intrinsic regulator of HSC function, stress response, and aging. However, little is known for its extrinsic function in regulating normal and malignant hematopoiesis. In this study, we reported its cell-extrinsic regulatory role of leukemogenesis. We found that *Lxn* deletion in BM niche enhances the survival of MLL-AF9–induced AML mice by suppressing leukemia stem cells (LSCs) and blood blasts. Single-cell RNA-seq (scRNA-seq) of stromal cells and cell communication analysis uncovered downregulation of the leukemia inhibitory factor receptor (LIFR) signaling pathway in the *Lxn*^–/–^ niche. The low LIFR level in *Lxn*^–/–^ MSCs upregulates *Cxcl9* expression, increasing CD8 T cells recruitment and cytotoxicity of leukemic cells. Combination of *Lxn* niche deletion and anti–PD-1 immune checkpoint inhibitor further increased survival. The findings advance our understanding of how niche regulates leukemogenesis and provide a potential treatment by *Lxn* inhibition in the niche to refine and improve current AML therapy.

## Results

### Lxn deletion alters cellular composition in BM niche.

The function of *Lxn* in regulating BM niche is unknown. We characterized the main niche cell populations, including mesenchymal stromal cell (MSC, CD45^–^Ter119^–^CD31^–^Sca1^+^CD51^+^), osteoblast (OB, CD45^–^Ter119^–^CD31^–^Sca1^–^CD51^+^), and endothelia cell (EC, CD45^–^Ter119^–^CD31^+^Sca1^+^) by flow cytometry in WT and *Lxn*^–/–^ mice (Figure 1A) (14, 42, 43). The percentages and absolute numbers of MSCs and OBs significantly increased in the *Lxn*^–/–^ niche compared with WT mice (Figure 1, B and C), whereas EC population didn’t show significant differences (data not shown). The increased number of MSCs was further confirmed by CFU-F assay (Figure 1D). MSCs are important to maintain normal HSC function (44, 45). We previously reported that *Lxn*^–/–^ HSCs had increased capacity of self-renewal and regeneration (38). To further determine whether *Lxn* regulates HSC and hematopoiesis in a cell-extrinsic manner, we generated MSC-specific *Lxn*-KO mouse model by crossing Lxn^loxp/loxp^ line (Supplemental Figure 1A; supplemental material available online with this article; https://doi.org/10.1172/jci.insight.199771DS1) with the Nes-Cre line (Nes-Cre: Lxn^loxp/loxp^) and analyzed the peripheral blood (PB) lineage differentiation and BM HSPC populations by flow cytometry and colony assay (38). Surprisingly, we didn’t see any significant changes in WBC numbers, lymphoid or myeloid differentiation (Supplemental Figure 1, B and C), BM cellularity, or numbers of HSPC subpopulations (Supplemental Figure 1, D–G). We also analyzed the MSC in Nes-Cre: Lxn^loxp/loxp^ mice and found a significant increase in both frequency and absolute numbers of immunophenotypic MSCs (Figure 1E). We made the OB-specific *Lxn*^–/–^ mouse lines (Osx-Cre: Lxn^loxp/loxp^) and performed the same hematopoietic and stromal analyses. We didn’t see any significant differences in hematopoiesis or OBs in Osx-Cre: Lxn^loxp/loxp^ line (Supplemental Figure 2). These results suggest that *Lxn* deletion altered niche cellular composition but has no functional effects on hemostatic hematopoiesis.

### Lxn deletion in BM niche enhances AML mice survival and suppresses leukemic cells.

The expansion of normal MSCs and OBs has been shown to inhibit leukemia development (7, 14). We then asked whether a *Lxn*-depleted niche could affect leukemia development. We established a MLL-AF9–induced (MA9-induced) AML model in *Lxn*^–/–^ and WT mice. Lineage depleted WT BM cells (Lin^–^) were transduced with MA9-GFP retrovirus and transplanted into lethally irradiated *Lxn*^–/–^ or WT control mice (Figure 2A). We found that *Lxn*^–/–^ recipient mice had a significant survival advantage over WT recipient mice; the median survival time was 61 days in *Lxn*^–/–^ recipients versus 35 days in WT recipients (Figure 2B). Consistently, the percentage of leukemic blasts (GFP^+^) in the PB (Figure 2C) and the absolute numbers of LSCs (GFP^+^Lin^–^ckit^+^CD34^dim^CD16/CD32^+^, leukemia granulocyte–macrophage progenitor (L-GMP) in both PB and BM (Supplemental Figure 3, A and B) were significantly decreased in *Lxn*^–/–^ recipient mice at 42 days after transplant (Figure 2, D and E, respectively). To determine whether the survival advantage observed in *Lxn*^–/–^ recipients resulted from impaired leukemia propagation or from microenvironmental effects during disease development, we performed secondary transplantation. AML cells isolated from primary WT and *Lxn*^–/–^ recipient mice were transplanted into secondary WT and *Lxn*^–/–^ recipients. No significant differences in leukemic burden were observed among the 4 groups (Supplemental Figure 3C). These findings suggest that *Lxn* deficiency within the recipient microenvironment does not durably alter the leukemia-propagating capacity of AML cells. Rather, the protective effect of *Lxn* deletion appears to be mediated primarily through modulation of the BM microenvironment during leukemia establishment and early progression.

Since MSCs and OBs are altered in normal *Lxn*^–/–^ niche, we used Nes-Cre: Lxn^loxploxp^ and Osx-Cre: Lxn^loxp/loxp^ as the recipients and used the same strategy to induce AML in these mice. The results show that Nes-Cre: Lxn^loxploxp^ recipients had a significantly higher median survival (73 days) than the litter-mate control group (51 days), while Osx-Cre: Lxn^loxp/loxp^ recipients showed a trend of survival advantage but without statistical significance (Figure 2F). Consistently, the percentage of GFP^+^ blast cells in PB were significantly lower in Nes-Cre: Lxn^loxp/loxp^, but not Osx-Cre: Lxn^loxp/loxp^ recipients, compared with the control group (Figure 2G). These data suggest that *Lxn* deletion in the BM niche, particularly within MSCs, reduces LSC and blast quantity, thus suppressing AML progression through microenvironment-dependent mechanisms. We therefore next investigated how *Lxn* deficiency alters stromal cell composition and stromal-leukemia communication networks.

### LIFR signaling is downregulated in Lxn–/– AML niche.

To further understand the cellular and molecular changes in *Lxn*^–/–^ AML niche, we performed the scRNA-seq on stromal and hematopoietic cells from WT and *Lxn*^–/–^ mice under homeostatic (non-AML) and AML conditions. Stromal cells were sorted as CD45^–^, Lin^–^, Ter119^–^, CD71^–^, and 7-AAD^–^ population, while hematopoietic cells were Lin^–^ in the non-AML condition and GFP^+^ in the AML condition. After quality control, around 12,000 cells were sequenced in each sample. Merging all samples and unsupervised clustering revealed distinct stromal and hematopoietic populations (Figure 3A). Under AML conditions, dominant monocytic blast populations largely replaced the normal hematopoietic hierarchy (Figure 3B and Supplemental Figure 4A). In parallel, AML profoundly remodeled the BM niche, with MSC1 emerging as the predominant mesenchymal population and replacing fibroblasts as a major stromal component (Figure 3B).

To determine the molecular mechanisms, we identified differentially expressed genes in various stromal clusters under normal and AML conditions (Supplemental Tables 1–4). To investigate potential communication networks between stromal cells and leukemic cells, we performed CellChat analysis on the scRNA-seq dataset. The inferred cell-cell communication network revealed that MSC1 exhibited the highest predicted interaction probability, with leukemia blasts among the stromal cell populations analyzed (Figure 3C). In our dataset, MSC1 was characterized by high expression of canonical mesenchymal niche genes that support hematopoiesis, including *Cxcl12* and *Lepr*, together with moderate expression of *Pdgfra* (Supplemental Figure 4B). This observation is consistent with our transplantation studies (Figure 2F), suggesting an important role for MSC in supporting leukemia progression. Comparison of signaling pathway activity between WT and *Lxn*^–/–^ niches further identified several receptor/ligand pathways, including Oncostatin M (OSM), vitronectin (VTN), and LIFR signaling, that displayed substantially higher information flow in the WT condition than in the *Lxn*^–/–^ condition (Figure 3D). The results suggest that these pathways may contribute to leukemia-supportive interactions within the WT BM microenvironment, whereas *Lxn* deletion remodels stromal-leukemia communication networks and reduces the activity of these pathways.

OSM, a member of IL-6 family, plays an important role in inflammation (46). GSEA of WT and *Lxn*^–/–^ AML niches revealed the downregulation of IL-6 pathways in the *Lxn*^–/–^ AML niche (Supplemental Figure 4C). OSM signals primarily through two types of receptor complexes: the type I receptor, a heterodimer of GP130 and LIFR, and the type II receptor, a heterodimer of GP130 and OSMR (46, 47). We examined the expression of these receptors in each stromal cluster and found that only *Lifr* was significantly downregulated in the MSC1 cluster (Supplemental Tables 1–4). To validate these findings, we performed qPCR using cultured MSCs according to our previously published protocol (48). We first evaluated whether cultured MSCs share features with the MSC1 population. We examined the expression of key MSC marker genes identified in our scRNA-seq dataset, including *Sca1*, *Cxcl12*, *Lepr*, and *Pdgfra*, by qPCR. Cultured MSCs expressed high levels of *Cxcl12* and *Sca1*, moderate levels of *Pdgfra*, and very low levels of *Lepr* (Supplemental Figure 4D). These findings indicate that high *Cxcl12* expression and moderate *Pdgfra* expression are common features shared by MSC1 and cultured BM-MSCs. Compared with WT MSCs, *Lxn*^–/–^ MSCs exhibited significantly reduced expression of *Lifr* and *Gp130*, while the expression of other LIFR signaling components, including *Osmr*, was unchanged (Figure 4A). We further assessed the protein expression of these receptors and found that only LIFR expression was decreased in *Lxn*^–/–^ MSCs, whereas GP130 and OSMR expression remained unchanged (Figure 4B). These results suggest a downregulation of LIFR signaling in the *Lxn*^–/–^ niche. LIFR signals through downstream JAK/STAT3, MAPK/ERK, and PI3K/AKT pathways (49–51). We found that p-STAT3 and p-ERK expression was decreased in *Lxn*^–/–^ MSCs (Figure 4, C and D). Taken together, these data suggest that *Lxn* deletion leads to the downregulation of LIFR signaling in MSCs, which suppresses the leukemia development.

### LIFR downregulation enhances immune surveillance in the AML Lxn^–/–^ niche.

LIFR signaling activation promotes cancer progression and is associated with poor prognosis in multiple malignancies (50, 51). In addition, LIFR signaling has been implicated in shaping the immune landscape of AML through regulation of T cell responses, macrophage function, and cytokine production. We therefore investigated whether *Lxn* deletion alters immune and cytokine profiles within the AML BM niche. Flow cytometric analysis revealed a significant increase in both percentages and numbers of CD8^+^ and CD4^+^ T cells in the BM of *Lxn*^–/–^ AML mice compared with WT AML controls (Figure 5, A and B). CD8^+^ T cells are key mediators of antileukemia immunity through their ability to directly eliminate leukemic cells. To further assess the functional status of T cells, we examined PD-1 expression on CD8^+^ effector and memory T cell subsets. Notably, PD-1 expression was significantly reduced on CD8^+^ effector T cells from *Lxn*^–/–^ AML mice compared with WT AML mice (Figure 5C), suggesting a less exhausted and potentially more effective antileukemia T cell response in the *Lxn*^–/–^ niche. We examined the frequencies of neutrophils, macrophages, dendritic cells, NK cells, and B cells and found that they were comparable between WT and *Lxn*^–/–^ AML mice (Supplemental Figure 5A). Furthermore, no significant differences in percentages of CD8^+^ and CD4^+^ T cells were observed between homeostatic (non-AML) WT and *Lxn*^–/–^ BM (Supplemental Figure 5B), suggesting that *Lxn* deletion specifically modulates T cell responses in the leukemic microenvironment.

LIFR signaling modulates immune responses by regulating chemokine expression (49, 50, 52). We thus performed a cytokine array on the BM fluid of WT and *Lxn*^–/–^ AML mice and identified several cytokines with altered expression (Figure 5D). Among them, CXCL9 emerged as the top candidate, as it is a downstream target of LIFR signaling and plays a role in modulating tumor immune microenvironment (52). Increased levels of CXCL9 in the Lxn^–/–^ AML niche were further confirmed by ELISA (Figure 5E). It has been reported that LIFR signaling promotes the binding of repressive histone marks, H3K27me3, at the *Cxcl9* promoter, thus suppressing its transcription (52). We hypothesized that downregulation of LIFR signaling in *Lxn*^–/–^ MSCs results in reduced H3K27me3 binding to the *Cxcl9* promoter, leading to increased *Cxcl9* expression. To test this, we performed the ChIP-qPCR in WT and *Lxn*^–/–^ MSCs using H3K27me3 antibody and found that less H3K27me3 bound to the *Cxcl9* promoter in *Lxn*^–/–^ MSCs (Figure 5F). This result further confirms that *Lxn* deletion causes downregulation of *Lifr*, which leads to *Cxcl9* upregulation. The increased level of *Cxcl9* facilitates the recruitment of T cells to the AML niche, thereby enhancing immune surveillance.

### Lxn deletion in the niche increases T cell–mediated cytotoxicity of AML cells via CXCL9.

Given the accumulation of T cells and the increased CXCL9 level in the *Lxn*^–/–^ AML niche, we hypothesize that CXCL9 promotes T cell recruitment, thus enhancing the cytotoxicity of AML cells. To test this, we established a transwell coculture system in which MSCs and MA9-AML cells were placed at the bottom, while CD8^+^ T cells were on the top (Figure 6A). *Lxn*^–/–^ MSCs significantly promoted the recruitment of CD8^+^ T cells compared with WT MSCs, as shown by the increased fold of CD8^+^ T cells migrating to the lower chamber (Figure 6B). Concomitantly, the number of AML cells was significantly reduced in the presence of *Lxn*^–/–^ MSC (Figure 6C), and their apoptosis was increased (Figure 6D). The result indicates that *Lxn*^–/–^ MSCs promote CD8^+^ T cell migration and enhances T cell–mediated cytotoxicity.

To determine whether this effect was mediated by CXCL9, we added either a CXCL9-neutralizing antibody or its receptor (CXCR3) inhibitor AMG 487 (a selective small-molecule CXCR3 antagonist) to the coculture system. Both treatments significantly reduced CD8^+^ T cell recruitment and abolished the enhanced AML killing effect of *Lxn*^–/–^ MSCs (Figure 6, E and F). These results demonstrate that *Lxn* deficiency in MSCs facilitates CXCL9-dependent recruitment and activation of CD8^+^ T cells, thereby promoting immune-mediated clearance of AML cells.

Our CellChat analysis identified LIFR signaling as one of the pathways significantly attenuated in the *Lxn*^–/–^ niche. To determine whether reduced LIFR signaling contributes to the enhanced immune response, we restored *Lifr* expression in *Lxn*^–/–^ MSCs before coculture with MA9-AML cells and CD8^+^ T cells. *Lifr* overexpression effectively reversed the *Lxn*^–/–^ phenotype, as evidenced by increased STAT3 phosphorylation (Figure 6G), reduced CD8^+^ T cell migration (Figure 6H), and increased AML cell survival (Figure 6I). Together, these findings identify reduced LIFR signaling as an upstream regulator of the CXCL9-dependent recruitment of CD8^+^ T cells and the AML-suppressive effects associated with Lxn deficiency.

### Lxn deletion in the niche further enhances the immunotherapy efficacy of AML.

AML cells themselves can upregulate PD-L1 and other inhibitory immune checkpoint molecules for immune evasion (53). Although immune checkpoint blockade has shown remarkable efficacy in several solid tumors, clinical studies of PD-1/PD-L1 inhibitors in AML have generally demonstrated limited activity when used as monotherapy (54). Nevertheless, emerging evidence suggests that combination strategies targeting both leukemia cells and the immunosuppressive BM microenvironment may enhance therapeutic efficacy. CD8^+^ effector T cells from *Lxn*^–/–^ AML mice exhibited significantly reduced PD-1 expression compared with WT AML controls, indicating altered regulation of immune checkpoint receptor expression within the leukemic microenvironment. Given the central role of the PD-1/PD-L1 axis in suppressing antitumor immunity, we next examined whether PD-1 blockade could further enhance antileukemia responses in the context of *Lxn*-deficient niches. We evaluated the therapeutic efficacy of combining anti–PD-1 treatment with *Lxn* niche deletion in AML mouse models. Mice transplanted with MA9-transduced LSCs were administered anti–PD-1 monoclonal antibody (CD279, clone RMP1-14; Bio X Cell) on days 21, 28, and 35 after transplantation at a dose of 200 μg per injection. The anti–PD-1 antibody binds PD-1 on T cells, blocking its interaction with PD-L1, thereby relieving PD-1–mediated inhibitory signaling and sustaining T cell activation and cytotoxic anti-leukemia responses. Percentages of GFP^+^ cells were measured on day 17 (before treatment) and day 42 (3 weeks after treatment) after transplantation and the mouse survival was observed (Figure 7A). The results show that combination of anti–PD-1 treatment with *Lxn* niche depletion led to the lowest level of leukemic blasts in the blood (Figure 7B). Additionally, weight loss was minimal (Figure 7C), and survival was significantly increased (Figure 7D). These findings suggest that *Lxn* deletion in the niche improves the efficacy of AML anti-PD1 immunotherapy.

## Discussion

The BM niche plays a pivotal role in maintaining hematopoietic homeostasis and shaping the development of hematological malignancies, including AML. However, the molecular regulators within the niche that modulate both normal hematopoiesis and leukemogenesis remain largely unknown. In this study, we identified Lxn as a regulator of the BM microenvironment that influences both normal niche architecture and leukemia progression through cell-extrinsic mechanisms. *Lxn* loss leads to expansion of supportive stromal elements, suppression of oncogenic LIFR signaling, and increased T cell–mediated cytotoxicity via CXCL9 upregulation (Figure 7E).

Our initial characterization revealed that *Lxn* deletion led to a significant expansion of MSCs and OBs. Despite this altered stromal composition, hematopoietic differentiation and HSPC maintenance remained unaffected in MSC- or OB-specific *Lxn*-KO models, suggesting that *Lxn* loss alters niche composition without impairing steady-state hematopoiesis. These findings highlight a separation between structural niche remodeling and functional hematopoietic disruption in physiological condition. Importantly, under leukemic stress, *Lxn*-deficient niches exhibited a pronounced antileukemic phenotype. Mice lacking *Lxn*-either globally or specifically in MSCs demonstrated significantly prolonged survival following MLL-AF9–induced AML transplantation. This survival advantage was accompanied by reduced leukemic burden and a decrease in LSC populations. To elucidate the mechanisms underlying this antileukemic effect, we performed scRNA-seq and CellChat analysis, revealing disrupted niche organization in AML and identifying LIFR signaling as a key pathway downregulated in the *Lxn*-deficient niche.

In *Lxn*^–/–^ niche or MSCs, reduced LIFR expression mediates multiple antileukemic effects. First, LIFR functions as a key receptor for proinflammatory cytokines in the IL-6 family. Upon activation, LIFR can drive the secretion of inflammatory cytokines through autocrine mechanism, thereby creating a microenvironment that favors leukemia progression. Our scRNA-seq data demonstrated that the IL-6 signaling pathway is downregulated in the *Lxn*^–/–^ AML niche, indicating that loss of *Lxn* attenuates the inflammatory signaling cascade, becoming less supportive of leukemic growth. Second, we observed that downregulation of LIFR reshapes the immune landscape within the AML niche by promoting the transcription of *Cxcl9*, a chemokine known to recruit cytotoxic T cells. *Lxn*^–/–^ MSCs enhance the migration and cytotoxic activity of CD8^+^ T cells toward AML cells in a CXCL9-dependent manner. Finally, LIFR/STAT3 signaling has previously been shown to stabilize PD-L1 expression, contributing to immune escape in the tumor microenvironment (55). In our study, we found that *Lxn*^–/–^ MSCs exhibit decreased phosphorylation of STAT3. Furthermore, combining niche-specific *Lxn* deletion with anti–PD-1 treatment significantly prolonged survival in AML-bearing mice compared with either intervention alone, suggesting that *Lxn* loss may potentiate immunotherapeutic efficacy. Taken together, *Lxn*-mediated downregulation of LIFR contributes to reshaping the AML niche by reducing inflammation, enhancing immune surveillance, and increasing sensitivity to therapeutic interventions.

Our lab previously reported the cell-intrinsic role for *Lxn* in the regulation of hematopoiesis. We showed that *Lxn* deletion enhances HSC self-renewal and survival under both physiological and stress conditions, including exposure to radiation, 5-fluorouracil (5-FU), and replicative stress (31, 32, 34–36, 38–40). However, the *Lxn*^–/–^ mice did not develop hematologic malignancies during aging process and after the stress over the long term, suggesting that *Lxn* deletion by itself is insufficient to initiate hematologic malignancy. In addition, in our unpublished studies, we overexpressed MLL-AF9 in WT and *Lxn*^–/–^ Lin^–^ cells and performed serial colony-forming assays. We did not observe enhanced expansion of MLL-AF9–transduced *Lxn*^–/–^ cells compared with transduced WT controls, further suggesting that *Lxn* deletion does not intrinsically promote the outgrowth of leukemia-initiating cells. In this study, we demonstrate that *Lxn* deletion in the BM niche increases the numbers of MSCs, without disrupting normal hematopoiesis. More importantly, *Lxn* deletion in the niche also significantly suppresses leukemia development. Thus, the published and current findings suggest that targeting *Lxn* may offer a dual therapeutic advantage: enhancing normal HSC function and resilience to therapy-induced hematopoietic toxicity, while concurrently suppressing leukemogenesis. *Lxn* may represent a promising candidate for therapeutic intervention in cancer treatment. Ongoing efforts to develop small-molecule inhibitors of LXN hold promise for translating these findings into therapeutic applications.

Although this study elucidates the extrinsic role of *Lxn* in regulating hematopoiesis, several important questions remain and merit further investigation. First, the mechanism by which *Lxn* depletion leads to downregulation of LIFR is not yet fully understood. Our previous work in hematopoietic cells demonstrated that LXN can bind to RPS3. In the absence of LXN, RPS3 may be released and either promote the nuclear translocation of the NF-κB complex to activate gene transcription or facilitate proteasomal degradation of its binding partners (39). We speculate that the latter mechanism (RPS3-mediated degradation) may contribute to the reduction of LIFR level in the *Lxn*-deficient niche. Future studies will aim to clarify this regulatory pathway. Second, our current findings are based on the MLL-AF9–induced AML model. To determine whether changes in *Lxn* expression represent a broader phenomenon, it will be important to test additional AML or leukemia models. Given the observed reduction in inflammatory signaling in the *Lxn*^–/–^ niche, it is plausible that *Lxn* may have a more general role in modulating hematological diseases. Lastly, it remains unclear whether *Lxn* expression is altered in patients with AML and what clinical significance such changes may hold. Therefore, evaluating *Lxn* expression in BM stromal cells from patients with leukemia will be a critical next step to assess its potential as a diagnostic or prognostic biomarker, as well as a therapeutic target.

## Methods

### Sex as a biological variable.

In all mouse studies, both male and female mice were used. Sex was considered as a biological variable in the statistical analyses.

### Animals.

Nes-Cre mice (B6.Cg-Tg[Nes-Cre]1Kln/J, Stock No: 003771) and Osx-Cre mice (B6.Cg-Tg[Sp7-tTA,tetO-EGFP/cre]1Amc/J, Stock No: 006361) were both purchased from The Jackson Laboratory. Generation and validation of latexin constitutive KO (*Lxn*^–/–^) and conditional KO mice were previously reported (38). All mice used were 8–12 weeks old. For radiation, mice were exposed to a lethal (9 Gy) dose of total body irradiation in a Mark 1 irradiator (137 Cesium) (J.L. SHEPHERD & ASSOCIATES) at a rate of 1.0 Gy/min with attenuator, on a rotating platform.

### Immunostaining and flow cytometry.

BM cells were obtained from the femur by flushing the central cavity with 2% FBS HBSS. PB cells were lysed with red blood cell lysis buffer (Invitrogen, 00-4333-57). Single-cell suspensions were stained with fluorescence-conjugated antibodies in both analysis and sorting.

For analysis of leukemia granulocyte macrophage progenitor (L-GMP), BM or PB cells were stained with lineage antibodies, including CD5-APC-Cy7 (BioLegend, 100650, clone 53-7.3), CD3-APC Cy7 (BioLegend, CD8a-APC-Cy7 (BioLegend, 100714, clone 53-6.7), CD45R/B220-APC-Cy7 (BioLegend, 103224, clone RA3-6B2), CD11b/MAC-1-APC-Cy7 (BioLegend, 101226, clone M1/70), LY-6G/GR-1 APC-Cy7 (BioLegend, 108424, clone RB6-8C5), TER119/Ly-76 APC-Cy7 (BioLegend, 116223, clone TER-119), C-KIT-PE (BioLegend, 105808, clone 2B8), CD34-APC (BioLegend, HM34, clone 128612), CD16/CD32-PerCP-Cy5.5 (BioLegend, 156624, clone S17011E), and CD127-Pacific Blue (BioLegend, 351306, clone A019D5). Dendritic cells and NK cells were stained with CD45-BV510 (BioLegend, 103137, clone 30-F11), CD3e-PE/Cy7 (BioLegend, 152314, clone 500A2), CD11c-APC(BioLegend, 117310, clone N418), NK1.1-Pacific Blue (BioLegend, 108722, clone PK136), and B220-APC-Cy7. Neutrophils and macrophages were stained with CD45-BV510, CD11c-APC, GR1-APC-Cy7, CD11b-PerCP-Cy5.5, F4/80-Pacific Blue, and PD-1 (BD Biosciences, 568565, Cone 29F.1A12).

For normal BM and PB cells analysis, cells were stained with the antibody lineage cocktails (BD Biosciences, Biotin Rat Anti-Mouse CD5, 553019; BD Biosciences, Biotin Rat Anti-Mouse CD45R/B220, 553086; BD Biosciences, Biotin Rat Anti-CD11b, 553309; BD Biosciences, Biotin Rat Anti-Mouse CD8a, 553029; BD Biosciences, Biotin Rat Anti-Mouse Ly-6G and Ly-6C, 553125; BD Biosciences, Biotin Rat Anti-Mouse TER-119/Erythroid Cells, 553672), anti-Sca-1 (eBioscience, 25-5981-82), anti-cKit (BD Biosciences, 553356), anti-CD135 (BD Biosciences, 553842), anti-FcγR (CD16/32) (BD Biosciences, 560540), anti-CD127 (eBioscience, 48-1271-82), anti-CD34 (BD Biosciences, 553733), and Streptavidin (for lineage cocktails, BD Biosciences, 554063). PB lineage chimerism staining was antibody anti-CD45.1 (BD Biosciences, 558701), anti-CD45.2 (BD Biosciences, 561874), anti-B220 (for B cells) (BD Biosciences, 552094), anti-CD90.2 (for T cells) (eBioscience, 25-0902-82), anti-Gr-1 (BD Biosciences, 553128), and anti-CD11b (for myeloid cells) (eBioscience, 45-0112-82) (38).

For stromal cell identification, hematopoietic and nonhematopoietic cells, located in both perivascular (marrow) and endosteal niche (digested bones), were obtained based on our previously established method (42). Single-cell suspensions were washed and stained with antibodies CD45 (BD Biosciences, 559864), Ter 119 (BD Biosciences, 557909 [APC] or 557915 [FITC]), CD31 (BioLegend, 102422), CD51 (BD Biosciences, 551187), Sca-1 (eBioscience, 25-5981-82), Streptavidin (for Lepr, BD Biosciences, 554063), and 7-AAD (56). Flow cytometry was performed and analyzed on the BD LSR II and BD Symphony A3 Cytometers. Dead cells were excluded using 7-AAD (BioLegend, 420404), propidium iodide (PI) (BioLegend, 421301), or Zombie Aqua viability dye (BioLegend, 423102). Data were acquired on a FACSymphony flow cytometer and analyzed using FACSDiva software. Unstained cells were used to define gating thresholds.

### Colony forming unit-fibroblast (CFU-F) assays.

The CFU-F assay was performed using the MesenCult Expansion Kit (Mouse) (StemCell Technologies, 05513) as previously described (57). Briefly, 2 × 10^6^ BM nucleated cells were plated in 60 mm dishes and cultured at 37°C with 5% CO_2_. After 3 hours, nonadherent cells were removed. After 14 days, cultures were fixed and stained with Giemsa, and colonies were counted under light microscopy.

### Retrovirus transduction and transplantation of transduced leukemic cells.

MSCV-MLL-AF9–IRES-GFP retrovirus was generated as previously (58). Viral particles were produced in 293TA cells by calcium phosphate–mediated cotransfection with Gag/Pol and EcoEnv plasmids. Viral supernatants were collected between 36 and 72 hours, filtered, and titrated. Lineage^–^ BM cells were isolated from C57BL/6 mice using magnetic depletion and seeded onto RetroNectin-coated plates. Cells were transduced overnight in StemSpan SFEM (Stem Cell Technology) supplemented with Flt3L (100 ng/mL), SCF (50 ng/mL), IL-3 (20 ng/mL), TPO (20 ng/mL), and polybrene (8 μg/mL). In total, 1 × 10^6^ total cells (containing 1 × 10^5^ GFP^+^ cells) were transplanted into primary recipient mice. PB GFP^+^ chimerism was assessed starting at day 17 after transplantation. For secondary transplantation, 1 × 10^5^ GFP^+^ BM cells isolated from the first recipients (6 weeks after primary transplantation) were transplanted into secondary recipients, and PB and BM GFP blast cells and L-GMP were analyzed at 6 weeks after secondary transplantation. Animal survival was monitored in a blinded manner.

### scRNA-seq and CellChat analysis.

Single cells were barcoded using the 10× Chromium single-cell platform, and cDNA libraries were prepared following the manufacturer’s instructions (Chromium Single Cell 3′ Kits v3.1, 10× Genomics). BM cells were harvested from 5–10 WT or Lxn^–/–^ mice (either normal or AML mice). Stromal cells were sorted by cell surface marker CD45^–^, Lin^–^, Ter119^–^, and 7-AAD. Live cell counts were further determined using a hemocytometer. Libraries were sequenced on the NovaSeq 6000 (Illumina) platform (paired-end 150 bp). The sequencing reads were aligned to the mouse reference genome (mm10) and quantified using CellRanger (v5.0.0, https://www.10xgenomics.com) with default parameters. The filtered count matrix, features, and barcodes of each sample from CellRanger were imported into Seurat (v4.3.0) (59) to create Seurat objects. To accurately characterize the transcriptomic changes of BM and stromal cells at single-cell level in Lxn^–/–^ mice, the cells that meet the following criteria were removed: (a) cells with less than 1,000 or more than 7,500 detected features; (b) cells with mitochondrial transcripts that accounted for more than 10% of the total detected transcripts; (c) cells predicted to be doublets by Scrublet (v0.2.3) (60). Moreover, the genes detected in fewer than 3 cells for each sample were also excluded in the following analysis. All 8 samples (HSPCs and stromal cells before transplantation, GFP^+^ MLL-AF9 cells, and stromal cells after transplantation) were merged to perform principal component analysis (PCA) and dimensionality reduction analysis. Different HSPC and stromal populations were annotated with known lineage-specific and stromal markers (14, 61, 62).

Cell-cell communication analysis was performed with CellChat (63). CellChat models cell-cell communication by integrating a curated ligand-receptor interaction database (CellChatDB) with a mass-action-based probabilistic framework to quantify signaling strength between cell groups. It is widely used to infer intercellular communication networks from single-cell transcriptomic data, identify key signaling senders/receivers, and compare communication patterns across biological conditions. In this study, we extracted WT and Lxn^–/–^ cells separately from the Seurat object and created individual CellChat objects using createCellChat. These 2 objects were subsequently merged via mergeCellChat to enable direct cross-condition comparison. We then analyzed and visualized differences in cell-cell interaction strength between WT and Lxn KO conditions using the functions of compareInteractions, netVisual_heatmap, and rankNet.

### qPCR.

BM cells were plated in α-MEM containing 20% FBS and antibiotics. Nonadherent cells were removed after 72 hours. MSCs were expanded and used between passages 2–4. Total RNA was isolated using TRIzol reagent and reverse transcribed using a high-capacity cDNA synthesis kit. qPCR was performed using SYBR Green chemistry on an ABI 7500 Fast system. Relative expression was calculated using the 2^–ΔΔCt^ method with GAPDH as the internal control. Primer sequences for *Lifr*, *Lxn*, and *Gapdh* are: *Lifr* forward (F), 5′-AGCTCTGACCCTCCTGCAT-3′, reverse (R), 5′-TGGGTGACAAGAATGGAACCT-3′; *Lxn* F, 5′-GAAATCCCACCCACCCACTAT-3′, R, 5′-AGATGGTACTTGTGCCCTCTT-3′. *Gapdh* F, 5′-AGGTCGGTGTGAACGGATTTG-3′; R, 5′-TGTAGACCATGTAGTTGAGGTCA-3′. *Cxcl12* F, 5′-TGC ATC AGT GAC GGT AAA CCA-3′; R, 5′-CAC AGT TTG GAG TGT TGA GGA T-3′. *Pdgfra* F, 5′-AGA GTT ACA CGT TTG AGC TGT C-3′; R 5′-GTC CCT CCA CGG TAC TCC T-3′. *Lepr* F, 5′-GTC TTC GGG GAT GTG AAT GTC-3′; R, 5′-ACC TAA GGG TGG ATC GGG TTT-3′. *Sca-1* F, 5′-AGG AGG CAG CAG TTA TTG TGG-3′; *Sca-1* R, 5′-CGT TGA CCT TAG TAC CCA GGA-3′.

### Western blotting.

Cells were lysed in RIPA buffer (Sigma, R0278-50ML) supplemented with protease and phosphatase inhibitor cocktails (Thermo Fisher Scientific, 78446). Protein concentrations were determined using a BCA protein assay (Thermo Fisher Scientific, 23227), and equal amounts of protein were mixed with SDS sample buffer and denatured by heating. Protein samples were separated by SDS-PAGE and transferred onto PVDF membranes. Membranes were blocked with 5% nonfat milk in Tris-buffered saline containing 0.1% Tween-20 (TBST) for 1 hour at room temperature and then incubated with the indicated primary antibodies overnight at 4°C. After washing with TBST, membranes were incubated with the appropriate HRP-conjugated secondary antibodies for 2 hours at room temperature. Protein bands were visualized using an enhanced chemiluminescence detection system. β-Actin was used as a loading control. The following primary antibodies were used: LIFR (Proteintech, #22779-1-AP), STAT3 (Proteintech, #60199-1-Ig), phospho-STAT3 (Proteintech, #80199-2-RR), AKT (Cell Signaling Technology, #9272), phospho-AKT (Ser473) (Cell Signaling Technology, #9271), ERK1/2 (Cell Signaling Technology, #9102), phospho-ERK1/2 (Cell Signaling Technology, #9101), GP130 (Cell Signaling Technology, #3732), OSMR (Proteintech, #10982-1-AP), β-actin (Sigma-Aldrich, #A2228), anti-rabbit IgG, HRP-linked Antibody (Cell Signaling Technology#7074), anti-mouse IgG, and HRP-linked Antibody (Cell Signaling Technology #7076).

### ELISA and cytokine array.

CXCL9 protein levels in conditioned media were quantified using a mouse CXCL9 ELISA kit (RayBiotech, ELM-MIG-1) according to the manufacturer’s instructions. BM extracellular fluid was collected using an identical protocol from all mice. CXCL9 concentrations were determined from a standard curve and expressed as pg/mL. Equal volumes of BM fluid were analyzed. Cytokine profiles of BM fluid from WT and Lxn^–/–^ AML mice were assessed using a Proteome Profiler Mouse XL Cytokine Array (R&D Systems Inc.). Equal amounts of BM extracellular fluid were incubated with the cytokine array membranes according to the manufacturer’s protocol. Chemiluminescent signals were quantified using ImageJ (NIH) after background subtraction and normalized to the internal positive control spots on each membrane. Relative expression was calculated by setting the WT group as 1.

### T cell isolation, activation, and coculture.

Splenocytes were isolated from C57BL/6 mice, subjected to red blood cell lysis, and purified for naive CD8^+^ T cells using negative selection (CD8a^+^ T Cell Isolation Kit, mouse, BioLegend, 130-104-075). T cells were activated with anti-CD3/CD28 beads and cultured in complete medium supplemented with IL-2 (10 ng/mL). For coculture experiments, activated CD8^+^ T cells were seeded in transwell inserts above MLL-AF9–GFP (MA9-GFP) leukemic cells and MSC cells. Where indicated, cultures were treated with CXCR3 inhibitor AMG487 (1 μM) or anti-CXCL9 antibody (MCE 10 μg/mL).

### PD-1 antibody treatment to block PD-1/PD-L signaling.

Mice received i.p. injections of PD-1 antibody (200 μg, BioXcell, InVivoMAb anti-mouse PD-1 [CD279, BE0146]) on days 21, 28, and 35 after transplantation. Control mice received PBS.

### Statistics.

Data were assessed for homogeneity of variances using an F test and analyzed using a 2-tailed, unpaired Student’s *t* test, as appropriate. One-way or 2-way ANOVA was used for comparisons among multiple groups. Kaplan-Meier survival curves were compared using the log-rank (Mantel-Cox) test. The statistical analyses were performed using GraphPad Prism Software version 11. The results shown represent mean ± SD. Differences were considered significant at *P* < 0.05.

### Study approval.

Mice were housed at the University of Kentucky animal facilities and New York Blood Center animal facilities following NIH-mandated guidelines for animal welfare and with IACUC approval. All experimental procedures followed the approved IBC protocols.

### Data availability.

All scRNA-seq datasets were deposited in Gene Expression Omnibus database (GSE330528). The code used for scRNA-seq analysis is available at: https://github.com/PinpinSui/Lxn_Stromal (commit ID: 3993e4b76eb972b23aa8d67ff02546b8dc1fd48e). Values for all data points in graphs are reported in the Supporting Data Values file.

## Author contributions

CZ established the mouse models and performed all in vivo experiments. XY conducted all in vitro experiments and mechanistic studies. PS contributed to scRNA-seq analysis and data interpretation. CZ, XY, and PS contributed equally to this manuscript. The order of co–first authorship was determined based on the relative contributions to study design, experimental execution, data analysis, and manuscript preparation. LH and BY were involved in the bioinformatic and database analyses. LLL, GH, ST, YZ, and BS provided input in the leukemia mouse model and reviewed the manuscript. H Zheng and HQ provided clinic insights. H Zhong assisted with immune profiling. FCY supported the scRNA-seq, histopathological analysis, and manuscript review. YL supervised the overall project, designed the experiments, and wrote the manuscript.

## Conflict of interest

The authors have declared that no conflict of interest exists.

## Funding support

This work is the result of NIH funding, in whole or in part, and is subject to the NIH Public Access Policy. Through acceptance of this federal funding, the NIH has been given a right to make the work publicly available in PubMed Central.

- NIH (R01HL124015 and R21AI193881, YL).
- American Society of Hematology (ASH) Bridge Fund (YL).
- New York Blood Center (YL).

## Supplementary Material

Supplemental data

Supplemental data set 1

Supplemental data set 2

Supplemental data set 3

Supplemental data set 4

Unedited blot and gel images

Supporting data values

## Figures and Tables

**Figure 1:**
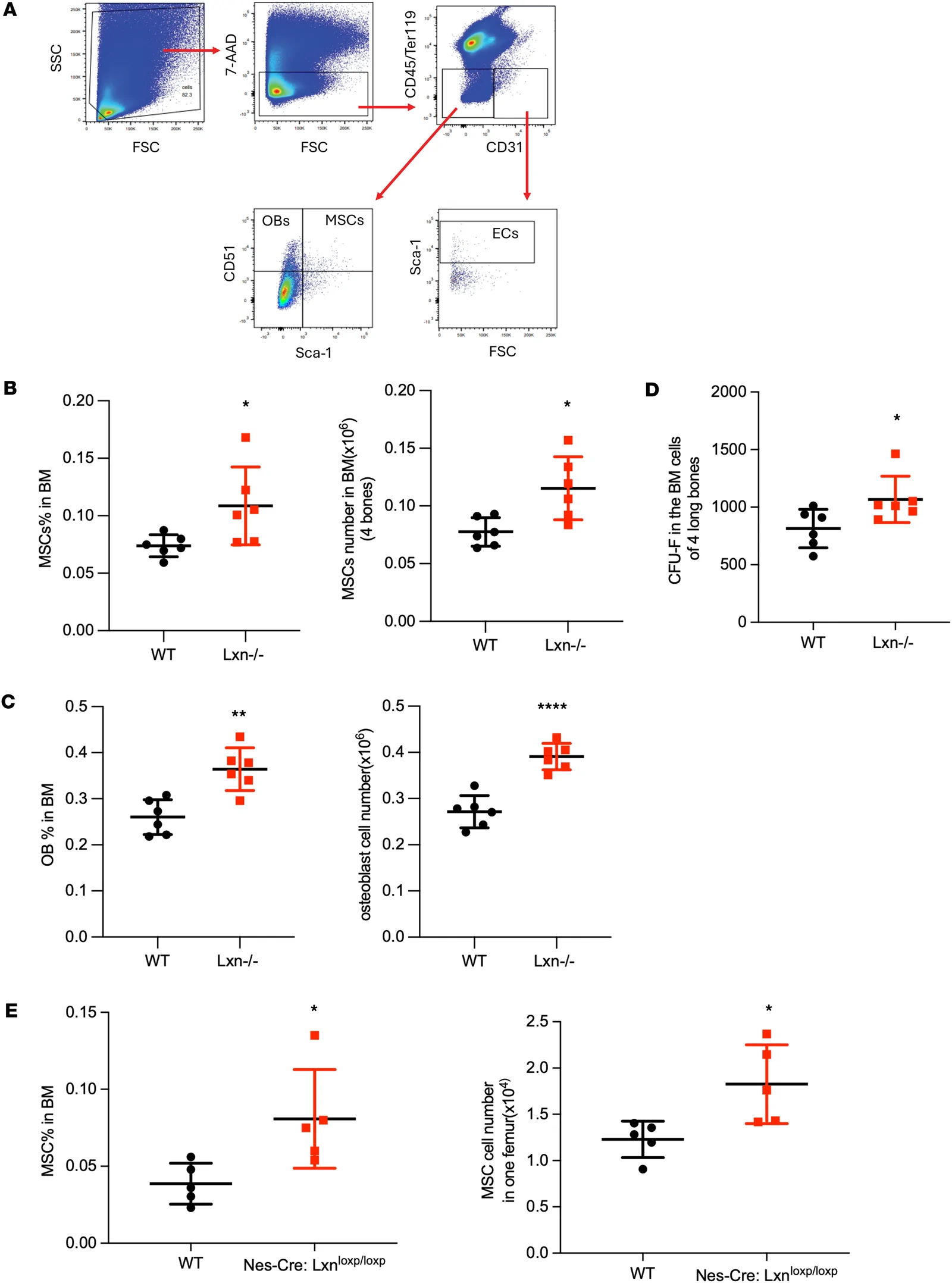
*Lxn* deletion alters BM niche cellular composition under steady-state conditions. (**A**) Representative flow cytometry profiles of MSCs, OBs, and endothelial cells (ECs). (**B**) Percentages (left panel) and absolute numbers (right panel) of MSCs in the *Lxn*^–/–^ mice compared with the WT mice. (**C**) Percentages (left panel) and absolute numbers (right panel) of OBs in the Lxn^–/–^ mice compared with the WT mice. (**D**) CFU-F numbers in the Lxn^–/–^ mice compared with the WT mice. (**E**) Percentages (left panel) and absolute numbers (right panel) of MSCs in the Nes-Cre: Lxn^loxp/loxp^ conditional KO mice compared with the WT litter-mate control mice. Data are shown as mean ± SD for the indicated number of mice from a single experiment. A 2-tailed, unpaired Student’s *t* test was performed. *P < 0.05, **P < 0.01, ****P < 0.0001.

**Figure 2:**
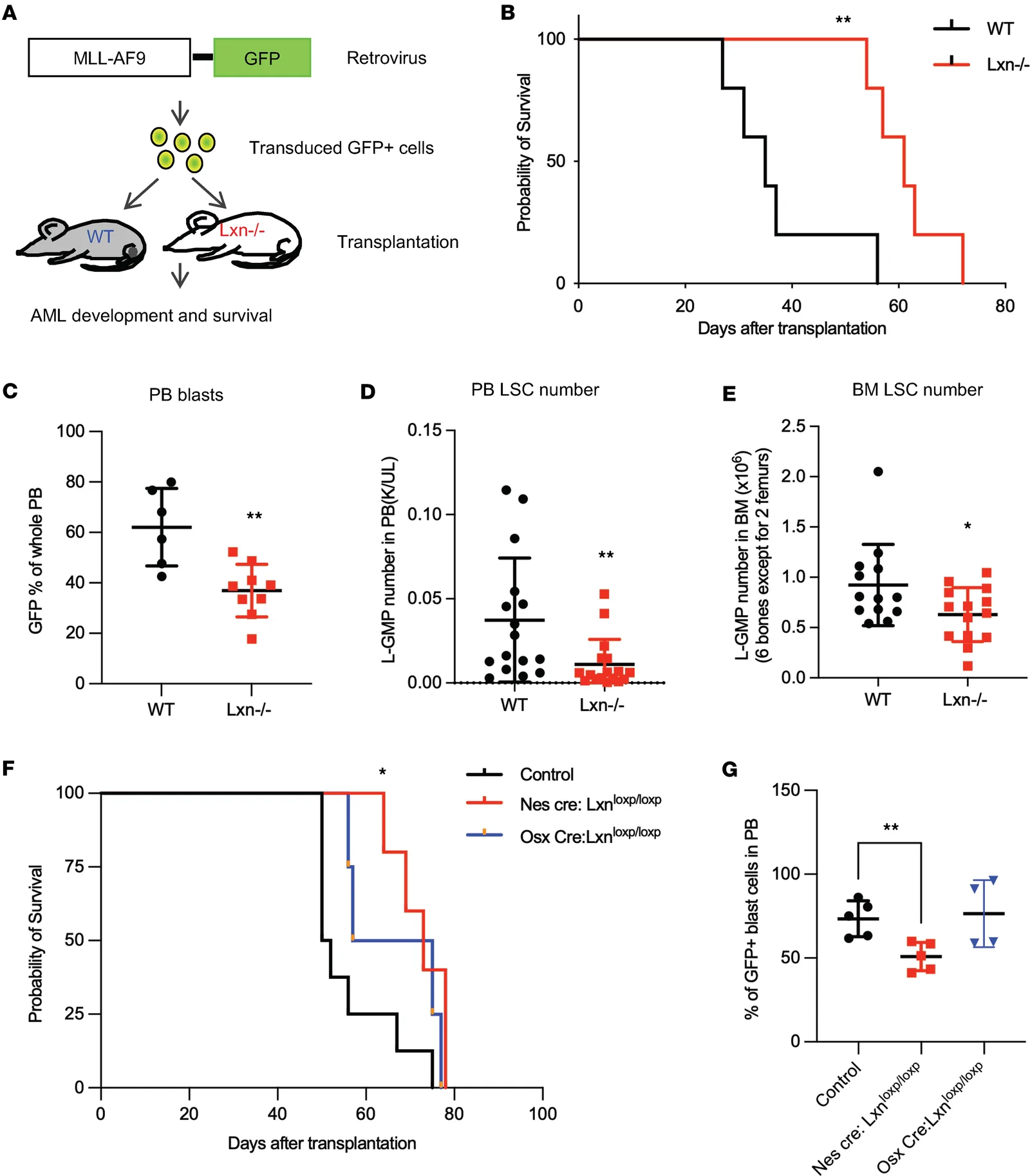
Lxn deletion in niche attenuates MLL-AF9–induced (MA9-induced) leukemia development. (**A**) Schematic diagram of MLL-AF9–induced AML development via retroviral transduction and transplantation into WT and Lxn^–/–^ recipient mice. (**B**) Kaplan-Meier survival curves of AML mice in Lxn^–/–^ or WT recipient mice. Statistical significance was determined using the log-rank (Mantel-Cox) test. (**C**) GFP^+^ leukemic blast percentages in peripheral blood (PB) of AML WT or Lxn^–/–^ recipient mice. Data are shown as mean ± SD for the indicated number of mice from 2 independent experiments. (**D** and **E**) Absolute number of leukemic stem cells (LSCs), identified as L-GMPs, in the PB and BM of AML WT or Lxn^–/–^ recipient mice. Data are shown as mean ± SD for the indicated number of mice from 3 independent experiments. (**F**) Kaplan-Meier survival curves of AML developed in the control, Nes-Cre; Lxn ^loxp/loxp^, or Osx-Cre; Lxn ^loxp/loxp^ conditional KO recipient mice. Overall differences were analyzed using the log-rank (Mantel-Cox) test, followed by pairwise comparisons with Holm-Šídák correction for multiple testing. (**G**) GFP^+^ leukemic blast percentages in PB of AML developed in the control, Nes-Cre; Lxn ^loxp/loxp^, or Osx-Cre; Lxn ^loxp/loxp^ conditional KO recipient mice. Data are shown as mean ± SD for the indicated number of mice from a single experiment. A 2-tailed, unpaired Student’s *t* test was performed in **C**–**E**. One-way ANOVA was used for comparisons among multiple groups in **G**. *P < 0.05, **P < 0.01.

**Figure 3:**
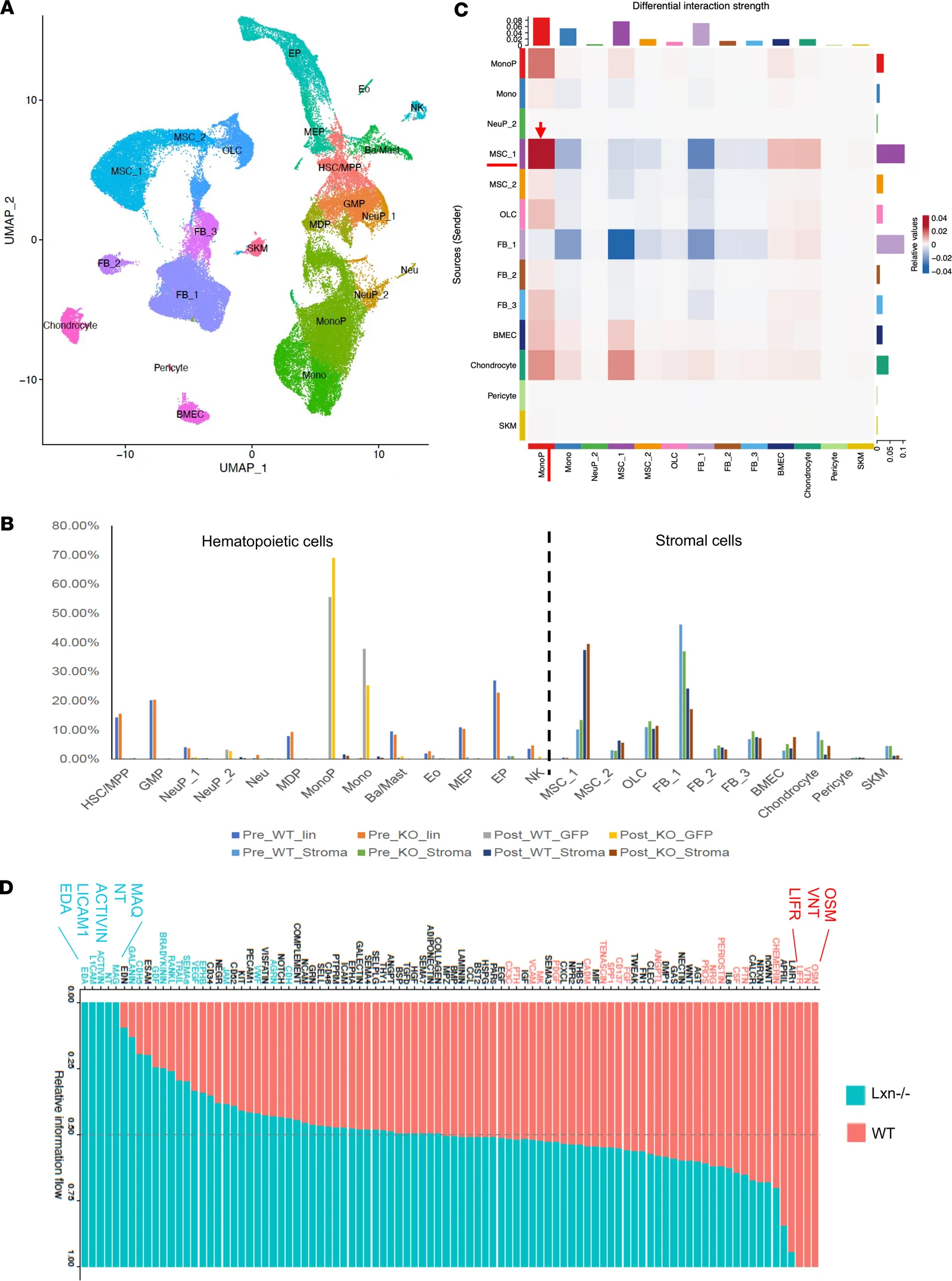
scRNA-seq on stromal and hematopoietic (or leukemic) cells from WT and Lxn^–/–^ homeostatic (non-AML) and AML mice. (**A**) The UMAP clustering of all 8 individual scRNA-seq samples on stromal and hematopoietic (or leukemic) cells from WT and Lxn^–/–^ (KO) homeostatic (non-AML) (Pre-) and AML (Post-) mice. Three distinct cell populations were identified, including stromal cells, hematopoietic cells, and MLL-AF9 blast cells. (**B**) The proportional distribution of hematopoietic (Lin^–^), leukemic (GFP), and stromal clusters in the BM of WT and Lxn^–/–^ (KO) homeostatic (non-AML) (Pre-) and AML (Post-) mice. (**C**) CellChat analysis reveals the differential interaction strength of stromal and leukemic cells between AML post-WT and post-KO cells. CellChat function *netVisual_heatmap* was used to visualize changes in cell-cell communication strength between WT and Lxn-KO samples. In this heatmap, the top bar represents the total incoming signaling strength (sum of column values) for each cell population, whereas the right bar represents the total outgoing signaling strength (sum of row values). Within the heatmap, red indicates increased interaction strength in the Lxn KO condition relative to WT, while blue indicates decreased interaction strength. The MSC1 population on the *y* axis and the MonoP blast population on the *x* axis are highlighted with red lines. Their interaction is indicated by the red arrow, representing the strongest cell-cell interaction. (**D**) Relative information flow comparison of ligand-receptor signaling pathways between WT and Lxn^–/–^ cells. We compared signaling pathway activity between WT and Lxn^–/–^ samples by calculating the information flow for each pathway, defined as the sum of communication probabilities across all interacting cell populations within the inferred signaling network. Signaling pathways were then ranked according to the difference in overall information flow between the 2 conditions. In the resulting plot, pathways with higher information flow in WT are displayed in red, whereas pathways with higher information flow in Lxn KO are displayed in green.

**Figure 4:**
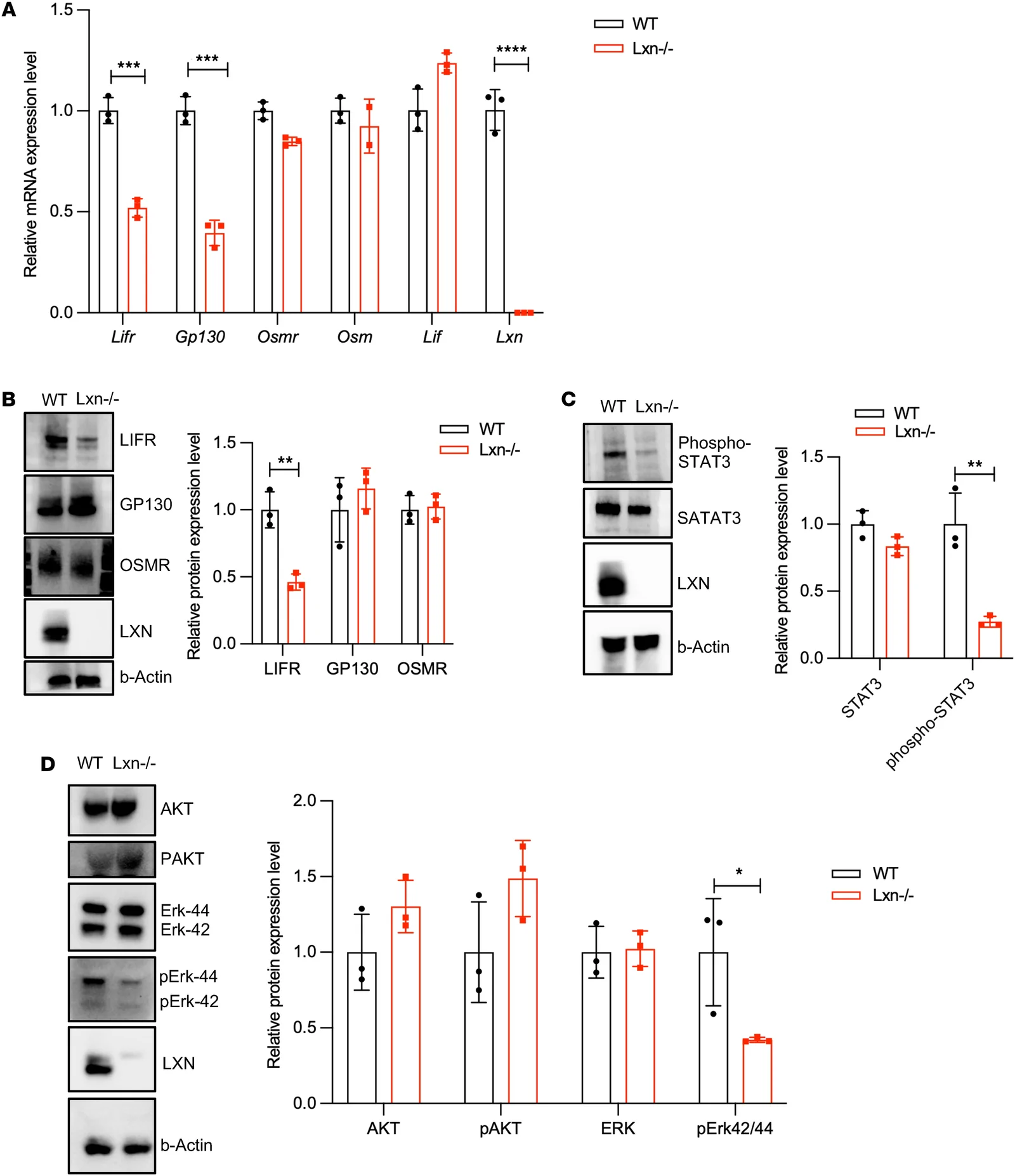
Lxn deficiency in MSCs downregulates LIFR signaling pathways. (**A**) qPCR analysis of mRNA expression levels of Lifr, Gp130, Osmr, Osm, Lif, and Lxn in cultured BM-derived MSCs from WT and Lxn^–/–^ mice. (**B**) Western blot of LIFR, GP130, and OSMR protein expression in cultured BM-derived MSCs from WT and Lxn^–/–^ mice. Densitometric quantification is shown on the right. Actin was used as a control. (**C**) Western blot of total STAT3 and phosphorylated STAT3 (p-STAT3) in cultured BM-derived MSCs from WT and Lxn^–/–^ mice. Densitometric quantification is shown on the right. Actin was used as a control. (**D**) Western blot of total and phosphorylated forms of AKT and ERK in cultured BM-derived MSCs from WT and Lxn^–/–^ mice. Densitometric quantification is shown on the right. Actin was used as a control. Data are shown as mean ± SD for the indicated number of replicates from a single experiment. A 2-tailed, unpaired Student’s *t* test was performed. *P < 0.05, **P < 0.01,***P < 0.001, ****P < 0.0001.

**Figure 5:**
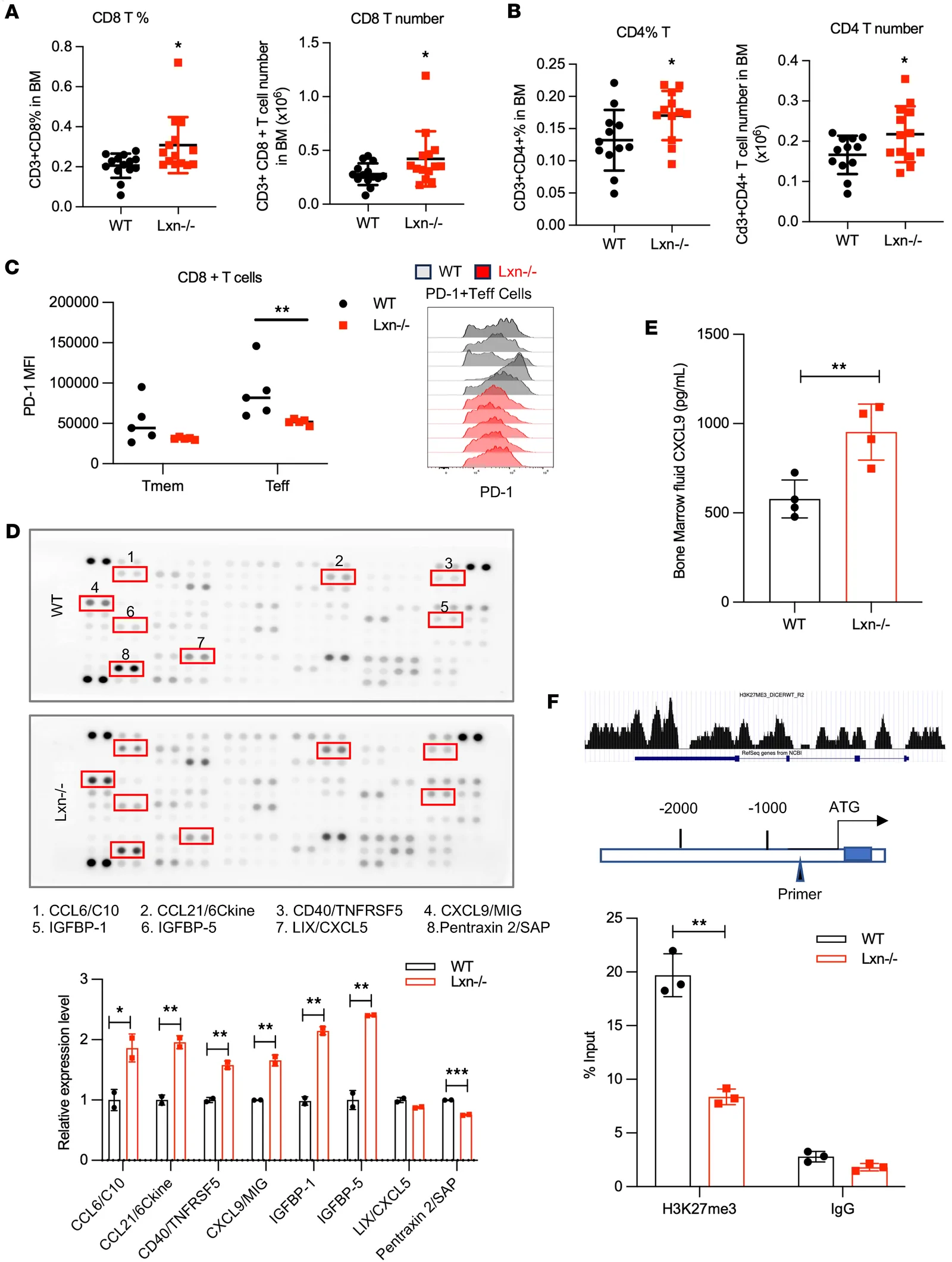
Lxn^–/–^ niche increases Cxcl9-mediated T cell accumulation. (**A**) Percentages (left panel) and absolute numbers (right panel) of CD8^+^ T cells in the BM of WT or Lxn^–/–^ AML mice. Data are shown as mean ± SD for the indicated number of mice from 3 independent experiments. (**B**) Percentages (left panel) and absolute numbers (right panel) of CD4^+^ T cells in the BM of WT or Lxn^–/–^ AML mice. Data are shown as mean ± SD for the indicated number of mice from 3 independent experiments. (**C**) PD-1 level (mean fluorescence intensity, MFI) in CD8^+^ T memory and effector cells (left panel) and representative flow cytometry profiles (right panel) in the BM of WT or Lxn^–/–^ AML mice. Data are shown as mean ± SD for the indicated number of mice from a single experiment. (**D**) Cytokine array analysis of BM fluid from WT and Lxn^–/–^ AML mice. Differentially expressed cytokines are highlighted (top panel). Quantification of signal intensities is shown on the bottom panel. Chemiluminescent signals were quantified using ImageJ after background subtraction and normalized to the internal positive control spots of each membrane. Relative expression was calculated by setting the WT group as 1. Data are shown as mean ± SD from a single experiment with a duplicate. (**E**) ELISA of CXCL9 in the BM fluid of WT and Lxn^–/–^ AML mice. Data are shown as mean ± SD for the indicated number of mice from a single experiment. (**F**) H3K27me3 binding at the Cxcl9 promoter region (UCSC Genome Browser, top panel). Schematic of primers used for ChIP-qPCR (middle panel). ChIP-qPCR using an H3K27me3 antibody shows reduced H3K27me3 occupancy at the Cxcl9 promoter in Lxn^–/–^ MSCs compared with WT MSCs (bottom panel). IgG served as a negative control. Data are shown as mean ± SD for the indicated number of samples from a single experiment. A 2-tailed, unpaired Student’s *t* test was performed. *P < 0.05, **P < 0.01, ***P < 0.001.

**Figure 6:**
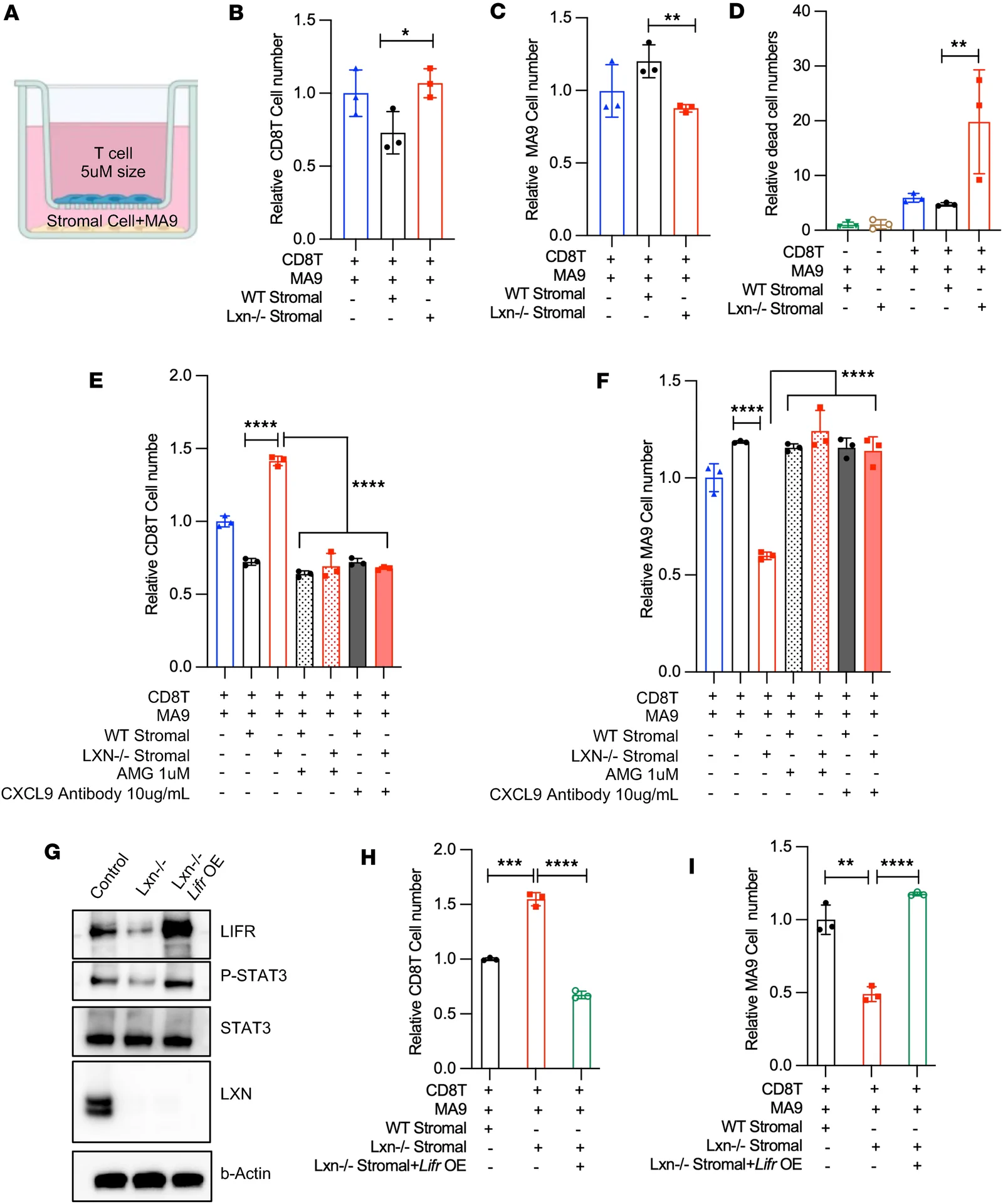
Lxn^–/–^ MSCs enhance CD8^+^ T cell–mediated cytotoxicity against AML cells through CXCL9. (**A**) Schematic representation of the transwell coculture system in which MSCs and MA9-AML cells were placed in the lower chamber, and CD8^+^ T cells were placed in the upper chamber. (**B**) Quantification of CD8^+^ T cells migrating into the lower chamber after coculture with WT or Lxn^–/–^ MSCs, demonstrating enhanced T cell recruitment by Lxn^–/–^ MSCs. (**C**) Relative MA9 AML cell number after coculture, showing reduced AML cell survival cultured with the Lxn^–/–^ MSCs. (**D**) Relative dead cells in CD8 T cells cocultured with WT or Lxn^–/–^ MSCs and MA9 cells, showing increased cell death in the Lxn^–/–^ MSCs. (**E** and **F**) Neutralization of CXCL9 or inhibition of CXCR3 signaling via AMG-487 (1 μM) in the transwell system abolished the enhanced CD8^+^ T cell recruitment (**E**) and AML cell killing (**F**) induced by Lxn^–/–^ MSCs. In **B**, **C**, **E**, and **F**, the number of migrated CD8^+^ T cells is presented as relative fold change normalized to the CD8 T and MA9-AML cells coculture group, which was set to 1. (**G**–**I**) Lifr was overexpressed in Lxn^–/–^ MSCs, and then cocultured with MA9-AML cells and CD8^+^ T cells. Lifr overexpression increased STAT3 phosphorylation (**G**), reduced CD8^+^ T cell migration (**H**), and increased AML cell survival (**I**). Data are shown as mean ± SD for the indicated number of samples from a single experiment. Comparisons among 3 or more groups were performed using 1-way ANOVA followed by Tukey’s multiple-comparisons test. *P < 0.05, **P < 0.01, ***P < 0.001, ****P < 0.0001.

**Figure 7:**
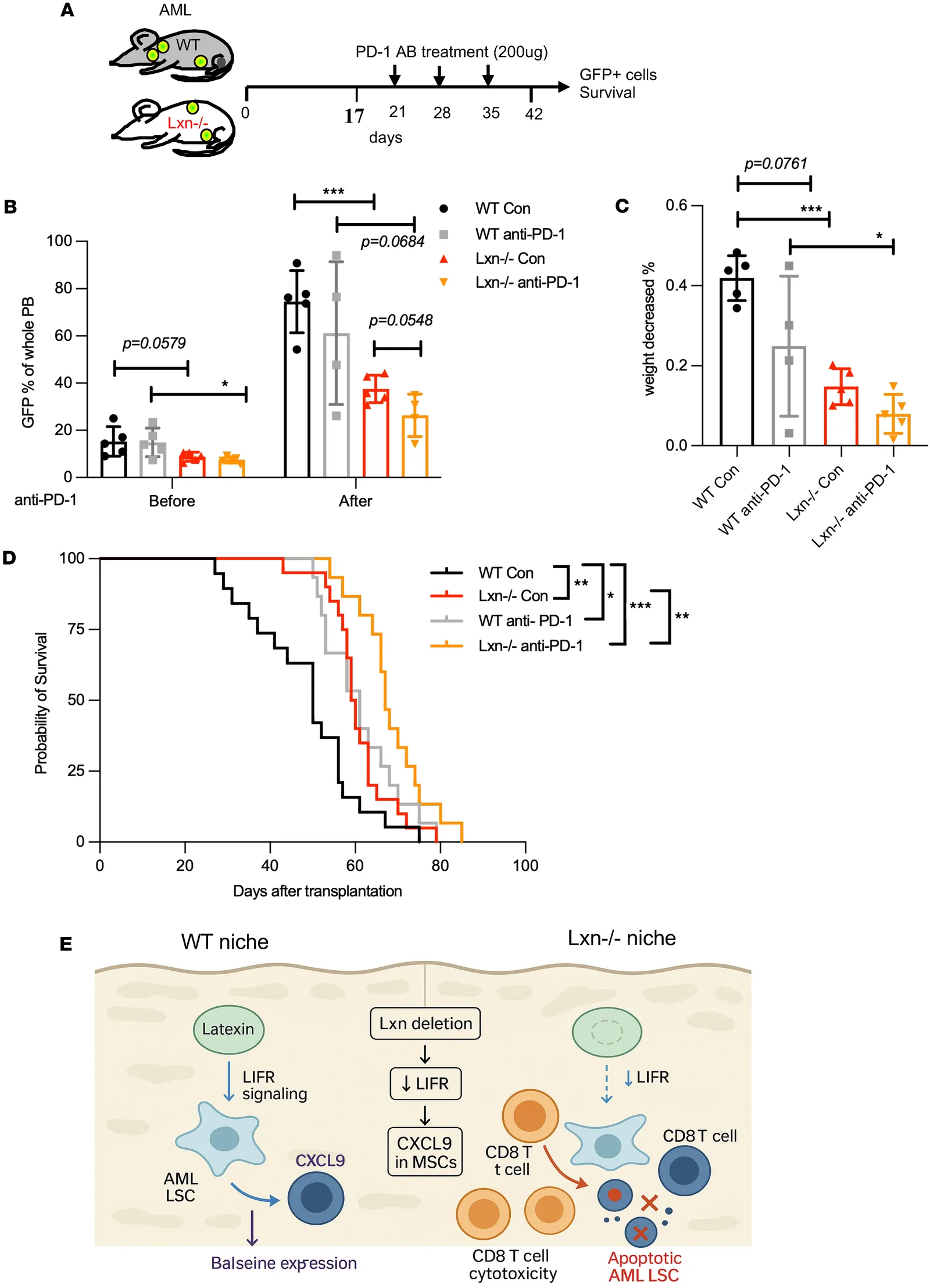
Lxn deletion in the niche enhances anti–PD-1 treatment efficacy of AML. (**A**) Experimental scheme for anti–PD-1 antibody treatment in MLL-AF9–driven AML mouse model. (**B**) Percentages of GFP^+^ leukemic blasts in PB at day 17 (pretreatment) and day 42 (posttreatment) after transplantation. Data are shown as mean ± SD for the indicated number of mice from a single experiment. (**C**) Weight loss of leukemic mice on day 42 after anti–PD-1 treatment compared with baseline. Data are shown as mean ± SD for the indicated number of mice from a single experiment. (**D**) Kaplan-Meier survival curve of leukemic mice with or without anti–PD-1 treatment. WT control (Con) group, *n* = 19; Lxn^–/–^ Con group, *n* = 20; WT anti–PD-1 group, *n* = 15; and Lxn^–/–^ anti–PD-1 group, *n* = 15. Overall differences were analyzed using the log-rank (Mantel-Cox) test, followed by pairwise comparisons with Holm-Šídák correction for multiple testing. Data are shown as mean ± SD for the indicated number of mice from 3 independent experiments. (**E**) Model of Lxn deletion in the BM niche and its effect on MLL-AF9 AML. Loss of Lxn downregulates LIFR signaling, leading to increased CXCL9 expression. Elevated CXCL9 recruits more cytotoxic CD8^+^ T cells, enhancing AML cell killing. Comparisons among 3 or more groups were performed using 1-way ANOVA followed by Tukey’s multiple-comparisons test. *P < 0.05, **P < 0.01, ***P < 0.001.

## Version 1 10/08/2026

Electronic publication
