# Supplementary material for: Latexin deletion in the bone marrow niche suppresses leukemia via LIFR downregulation and immune activation
Source: JCI Insight. 2026 Oct 8;11(19):e199771. doi: 10.1172/jci.insight.199771 (PMC13726419; doi:10.1172/jci.insight.199771)
Supplement: Supplementary file 1 [file jciinsight-11-199771-s012.pdf]

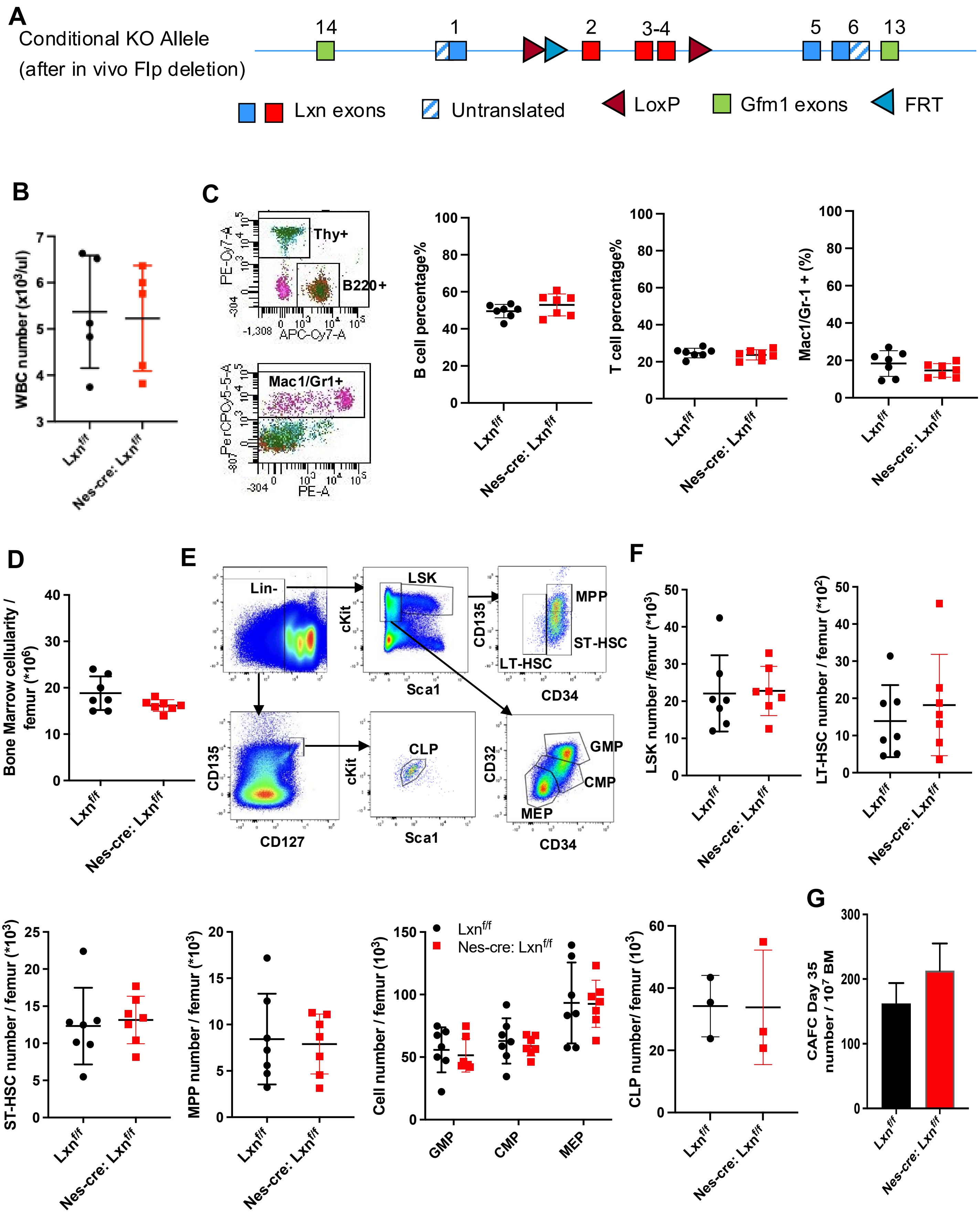

Supplement Figure 1

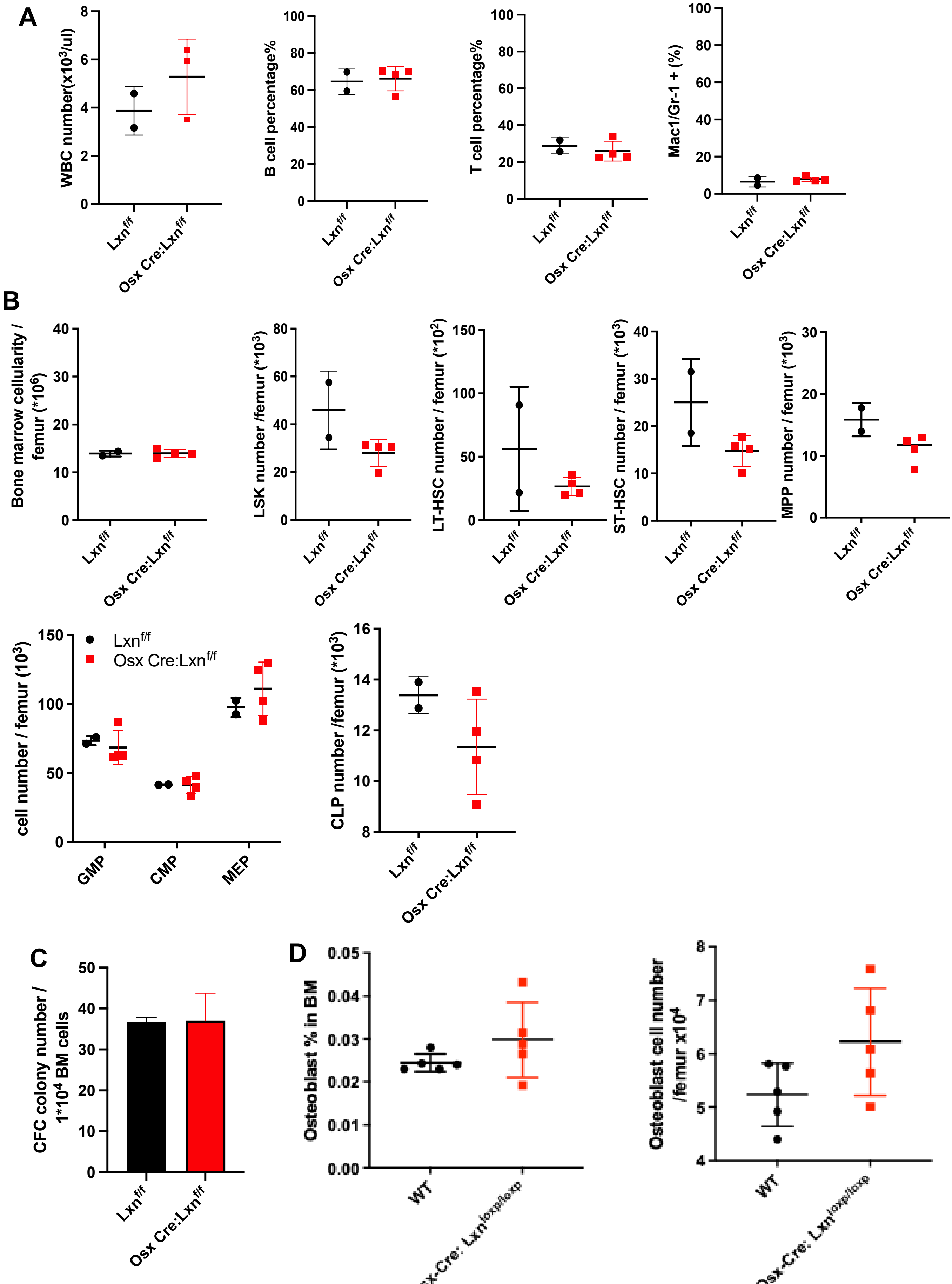

Supplement Figure 2

A

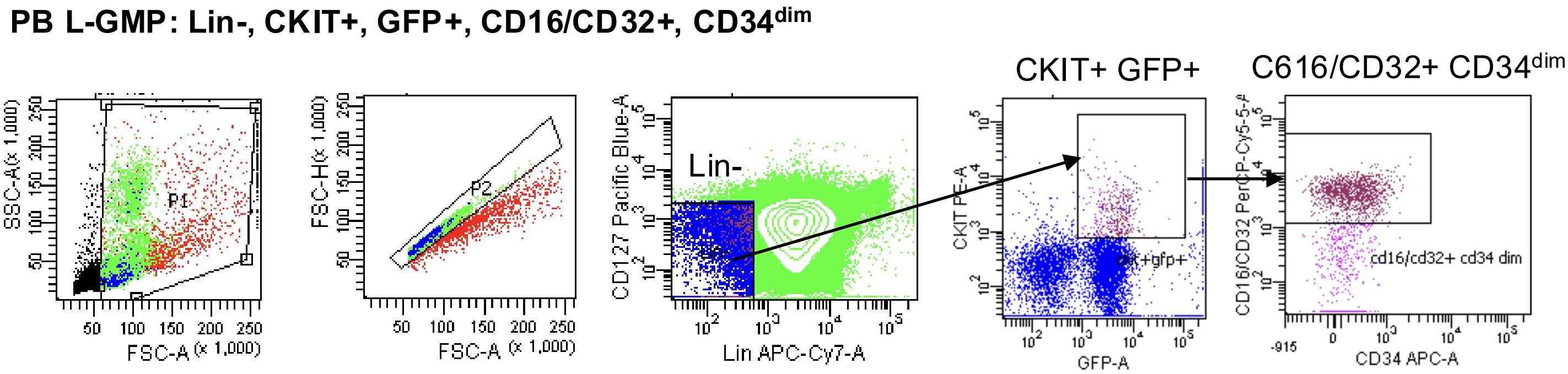

B

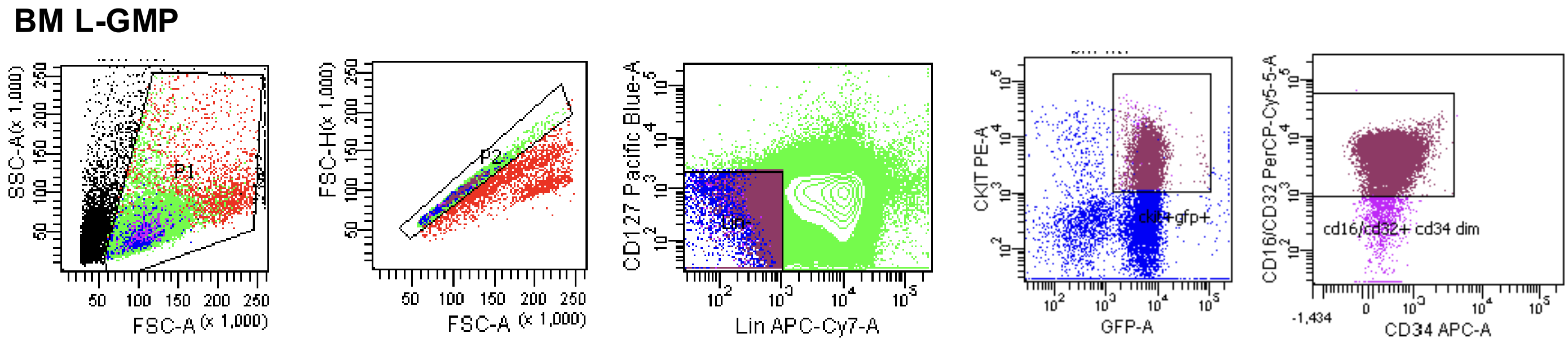

C

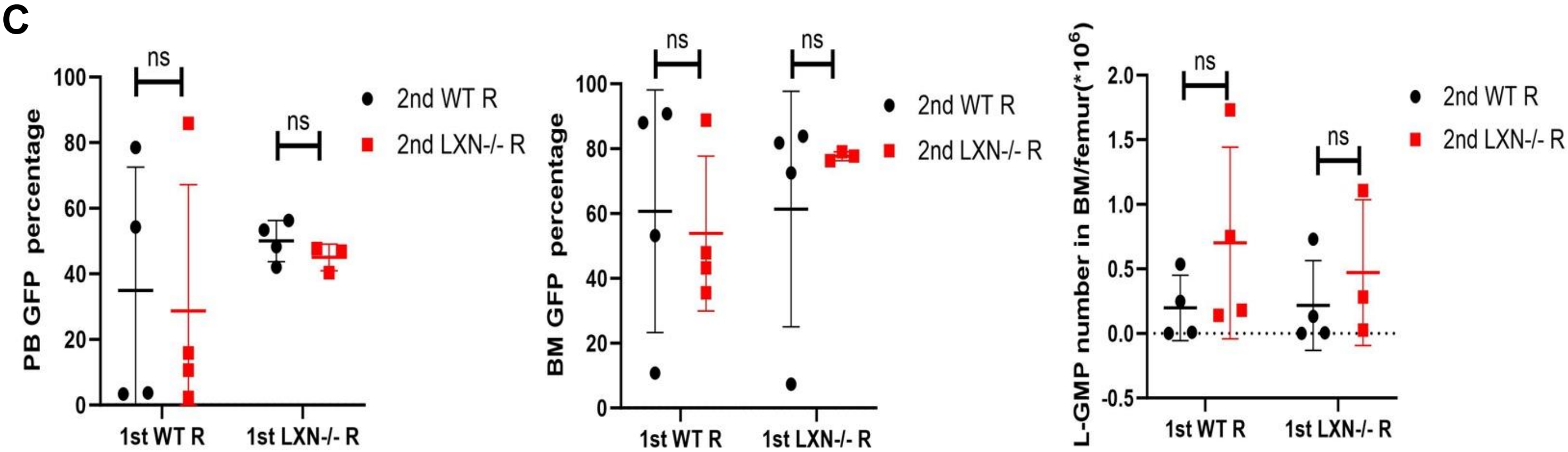

Supplement Figure 3

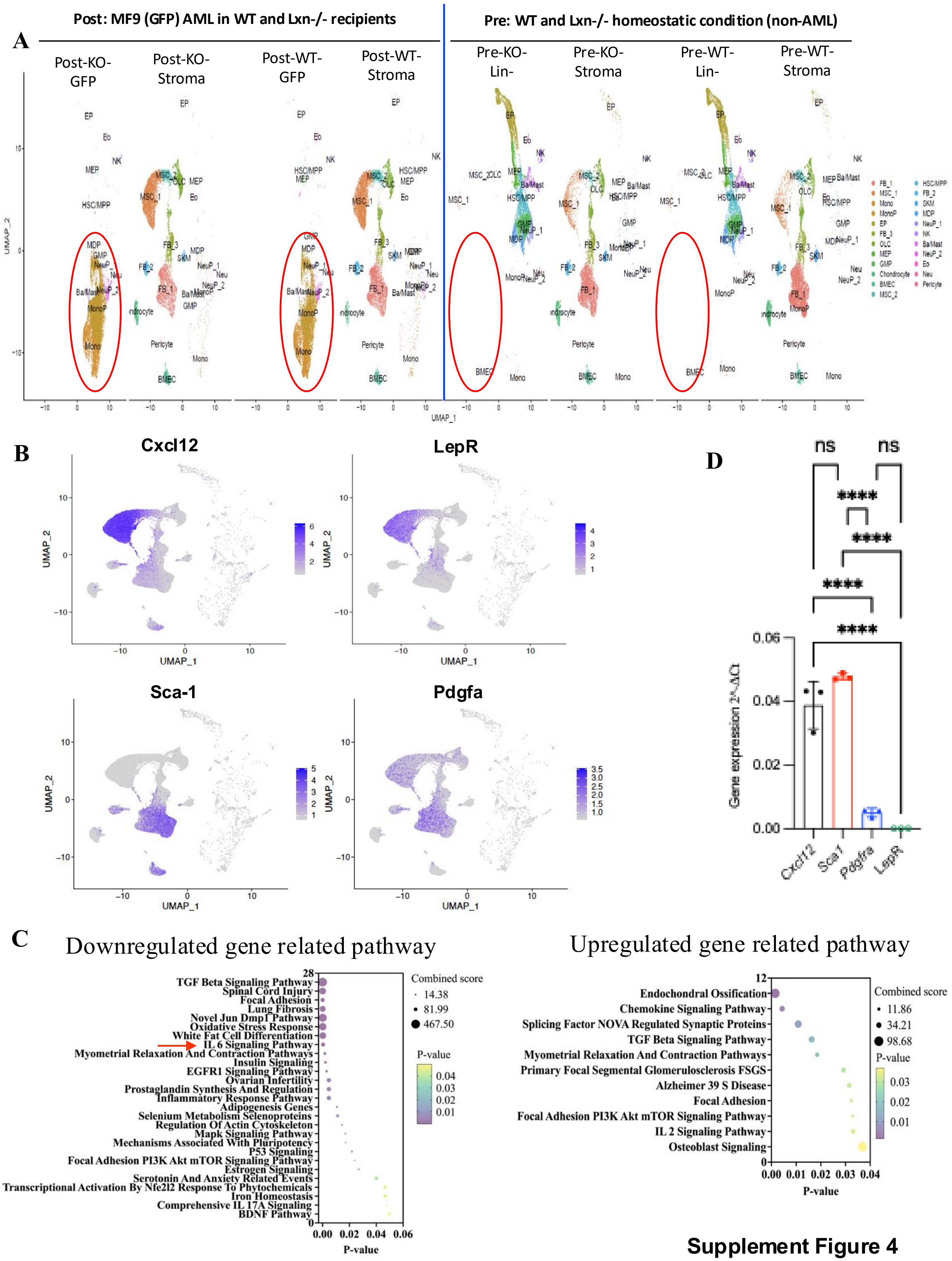

Supplement Figure 4

A

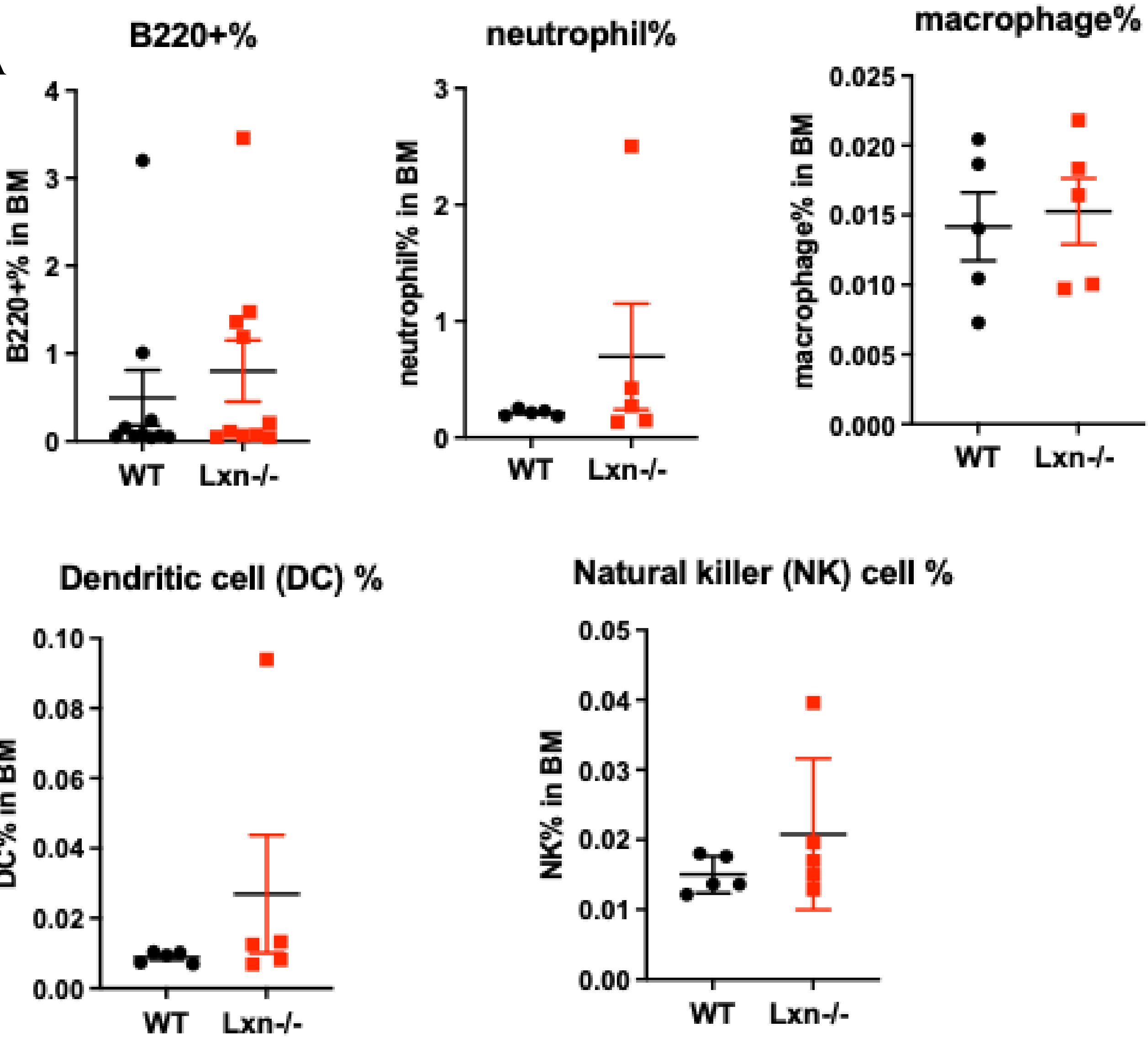

B

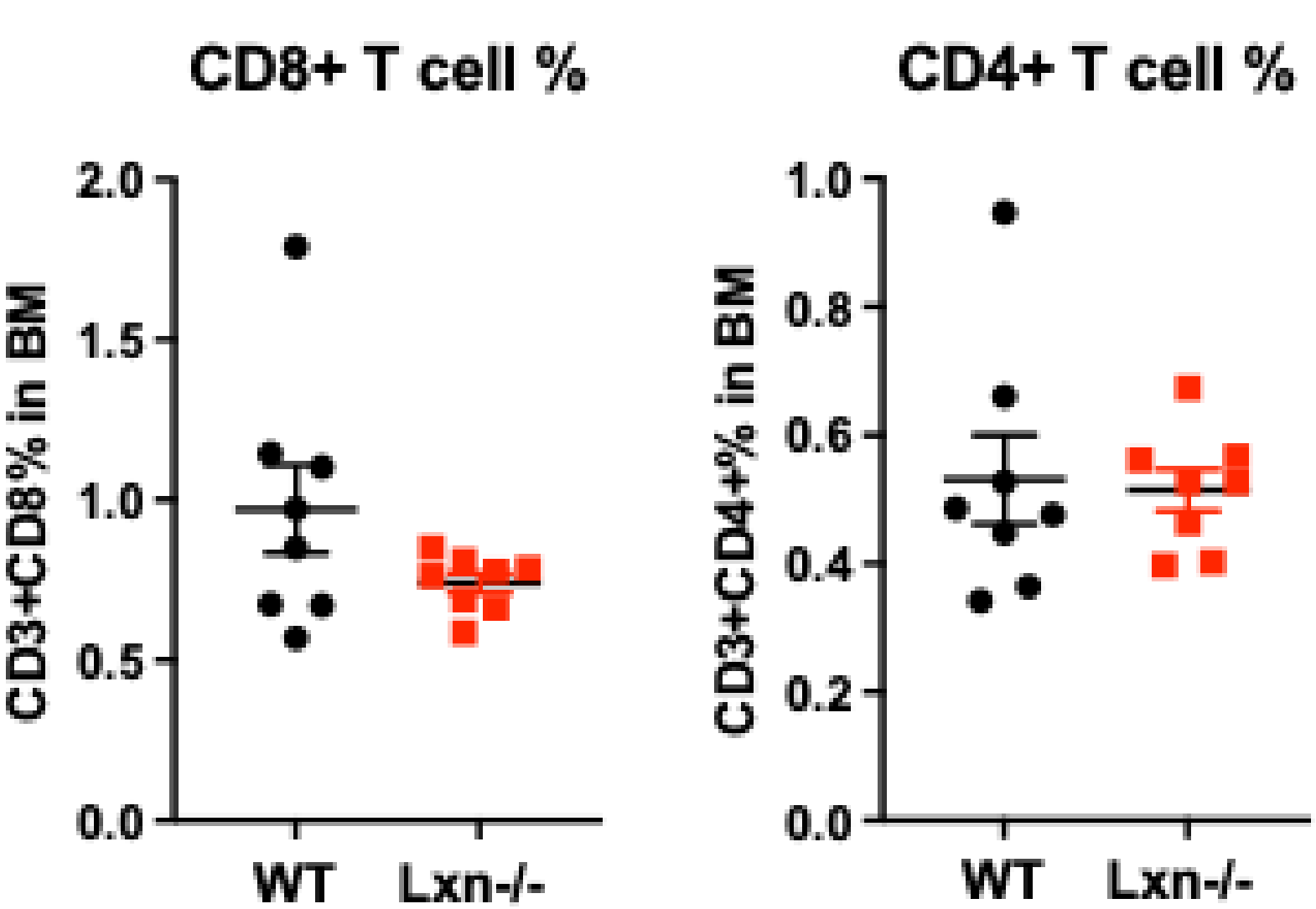

Supplement Figure 5

Supplementary Figure 1. Knockout Lxn in MSCs don't affect homeostatic hematopoiesis

(A) Schematic of the Lxn floxed allele ($Lxn^{f/f}$) and conditional knockout strategy. LoxP sites were inserted flanking exons 2-4 of the Lxn gene. Since Lxn resides within the Gfm1 gene, only exons 2-4 were targeted to minimize potential effects on Gfm1. Upon Cre induction, exons 2-4 of Lxn are excised, resulting in cell-specific gene deletion. **(B)- (G)** PB and BM hematopoietic analyses in the Nes-Cre:$Lxn^{f/f}$ conditional KO mouse compared to the litter-mate control ($Lxn^{f/f}$) control. No significant differences in the following cell populations: **(B)** white blood cell (WBC) counts; **(C)** flow-cytometry defined B (B220+), T (Thy1+), and macrophage and granulocytes (Mac1/Gr1+) cells; **(D)** BM cellularity; **(E)** flow cytometry-defined HSPCs, including **(F)** LSK cells, LT-HSCs, ST-HSCs, MPPs, myeloid committed progenitor cells (CMPs, GMPs and MEPs), and lymphoid committed progenitor cells (CLPs); and **(G)** functional clonogenic HSCs identified by cobblestone area forming cell (CAFC) at day 35 of culture. Values are presented as mean ± SD for the indicated number of mice from two independent experiments. A two-tailed, unpaired Student's t-test was performed.

Supplementary Figure 2. Knockout Lxn in OBs don't affect homeostatic hematopoiesis

PB and BM hematopoietic analyses in the Osx-Cre:$Lxn^{f/f}$ conditional KO mouse compared to the litter-mate control ($Lxn^{f/f}$) control. No significant difference in the follow cell populations: **(A)** white blood cell (WBC) counts and flow-cytometry defined B (B220+), T (Thy1+), and macrophage and granulocytes (Mac1/Gr1+) cells; **(B)** BM cellularity, and flow cytometry-defined HSPCs, including LSK cells, LT-HSCs, ST-HSCs, MPPs, myeloid committed progenitor cells (CMPs, GMPs and MEPs), lymphoid committed progenitor cells (CLPs), and **(C)** colony forming cell numbers. **(D)** The percentages and absolute numbers of OB cells didn't show difference in Osx-Cre:$Lxn^{f/f}$ conditional KO mouse. Values are presented as mean ± SD for the indicated number of mice from a single experiment. A two-tailed, unpaired Student's t-test was performed.

Supplementary Figure 3. Identification of L-GMP by flow cytometry and the effect of Lxn deletion in the niche on the L-GMP sustainability. Representative flow cytometry profiles of LSCs (L-GMP) in **(A)** peripheral blood and **(B)** BM of WT and Lxn-/- AML recipient mice. Cell surface markers were indicated. **(C)** Secondary transplantation experiments were performed to determine whether the survival advantage observed in Lxn-/- primary recipients resulted from impaired leukemia propagation or from microenvironmental effects during disease development. AML cells isolated from primary WT (1st WT R) and Lxn-/- recipient mice (1st Lxn-/- R) were transplanted into secondary WT (2nd WT R) and Lxn-/- recipients (2nd Lxn-/- R). No significant differences in leukemic burden in PB (left panel), BM (middle panel), and BM L-GMP (right panel) were observed among the four groups. Values are presented as mean ± SD for the indicated number of mice from a single experiment. One-way ANOVA was used for comparisons among multiple groups.

Supplementary Figure 4. Single-cell RNA sequencing of stromal and hematopoietic cells from WT and Lxn-/- mice before (pre) and after (post) MA9 cell transplantation. (A) UMAP clustering identifies distinct stromal and hematopoietic (or leukemic) cells in WT and Lxn-/- normal (non-AML) and AML recipients**.** Pre-WT and Pre-KO are the normal (non-AML) WT and Lxn-/- mice. Post-WT and Post-KO are AML WT and Lxn-/- recipient mice. **(B)** UMAP of key MSC marker genes (*LepR, Cxcl12, Ly6a and Pdgfra*) using merged scRNA-seq data. **(C)** Enriched signaling pathways up- and down-regulated in the Lxn-/- AML niche. Pathway enrichment analysis was performed on differentially expressed stromal cell genes from scRNA-seq of the Lxn-/- AML niche. Up- and down-regulated gene sets were analyzed using Enrichr (WikiPathways 2024 Mouse database). Significance was determined by combined score and adjusted P-value. Results are shown as dot plots, where dot size indicates combined score and color represents P-value. **(D)** Gene expression of MSC marker genes (*LepR, Cxcl12, Ly6a and Pdgfra*) in cultured MSCs measured by Quantitative PCR. Values are presented as mean ± SD

for the indicated number of replicates from a single experiment. One-way ANOVA was used for comparisons among multiple groups.

Supplement Figure 5. PB lineage analyses in the AML and normal WT and Lxn-/- mice.

(A) In BM of WT and Lxn-/- AML mice, no significant differences were observed in the percentages of B cells, neutrophils, macrophages, dendritic cells (DC) and natural killer (NK) cells. **(B)** In normal (non-AML) WT and Lxn-/- mice, no significant differences were observed in the percentages of **(B)** CD8 T cells (left panel) and CD4 T cells (right panel). Values are presented as mean ± SD for the indicated number of mice from a single experiment. A two-tailed, unpaired Student's t-test was performed.
