# Supplementary material for: Latexin deletion in the bone marrow niche suppresses leukemia via LIFR downregulation and immune activation
Source: JCI Insight. 2026 Oct 8;11(19):e199771. doi: 10.1172/jci.insight.199771 (PMC13726419; doi:10.1172/jci.insight.199771)

Figure4B

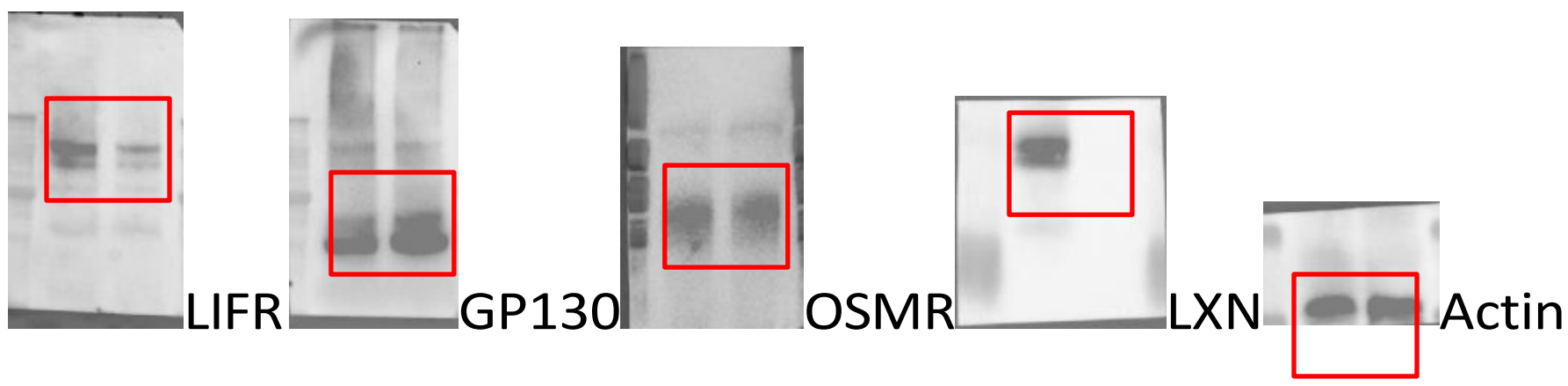

Figure4C

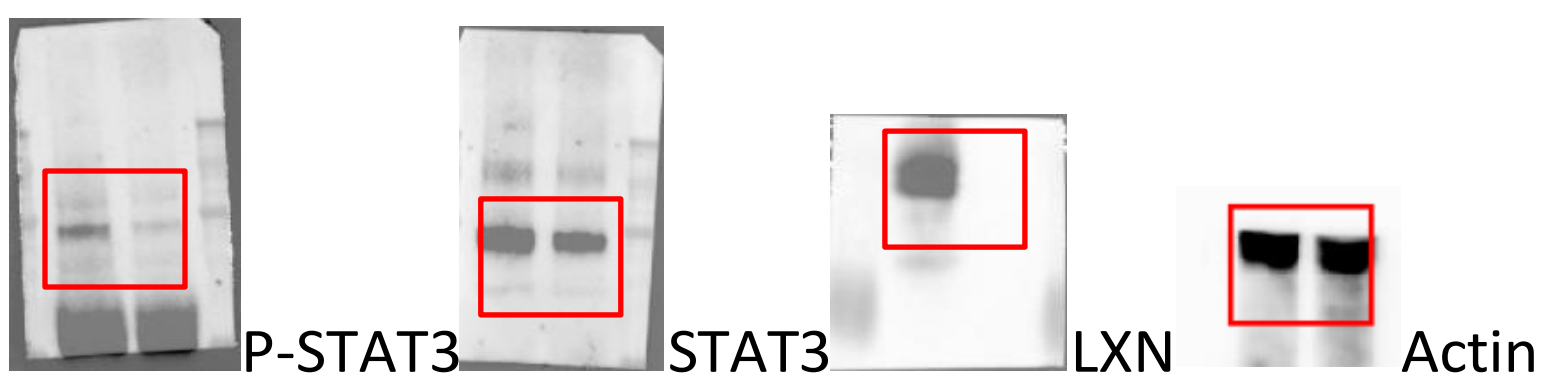

Figure4D

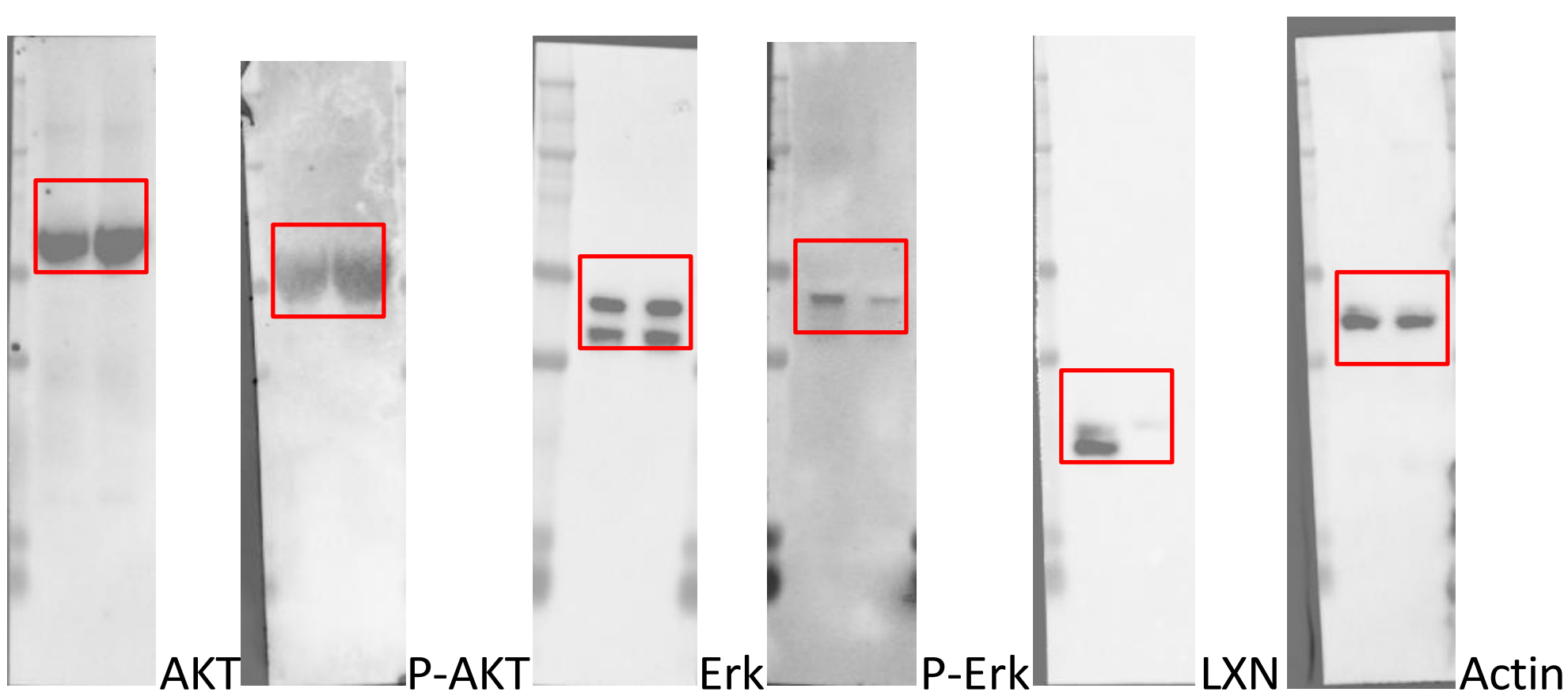

Figure6G

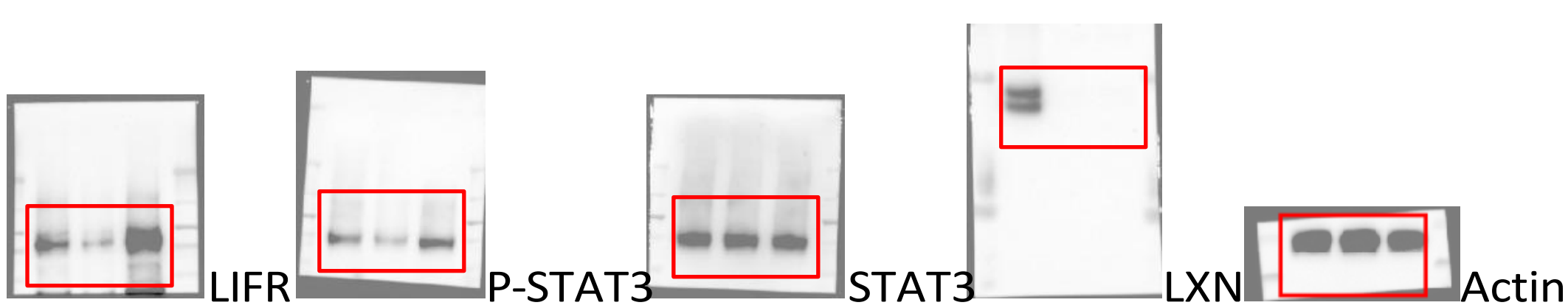

Supplement: Supplementary file 2 [file jciinsight-11-199771-s017.pdf]
